# Epithelial ovarian cancer is infiltrated by activated effector T cells co-expressing CD39, PD-1, TIM-3, CD137 and interacting with cancer cells and myeloid cells

**DOI:** 10.3389/fimmu.2023.1212444

**Published:** 2023-10-04

**Authors:** Elena Tassi, Alice Bergamini, Jessica Wignall, Miriam Sant’Angelo, Emanuela Brunetto, Chiara Balestrieri, Miriam Redegalli, Alessia Potenza, Danilo Abbati, Francesco Manfredi, Maria Giulia Cangi, Gilda Magliacane, Fabiola Scalisi, Eliana Ruggiero, Maria Chiara Maffia, Federica Trippitelli, Emanuela Rabaiotti, Raffaella Cioffi, Luca Bocciolone, Giorgio Candotti, Massimo Candiani, Gianluca Taccagni, Birgit Schultes, Claudio Doglioni, Giorgia Mangili, Chiara Bonini

**Affiliations:** ^1^ Experimental Hematology Unit, Division of Immunology, Transplantation and Infectious Disease, IRCCS Ospedale San Raffaele, Milano, Italy; ^2^ Cell Therapy Immunomonitoring Laboratory (MITiCi), Division of Immunology, Transplantation and Infectious Diseases, IRCCS Ospedale San Raffaele, Milan, Italy; ^3^ Università Vita-Salute San Raffaele, Milan, Italy; ^4^ Department of Obstetrics and Gynecology, IRCCS Ospedale San Raffaele, Milan, Italy; ^5^ Department of Surgical Pathology, IRCCS Ospedale San Raffaele, Milan, Italy; ^6^ Intellia Therapeutics, Inc., Cambridge, MA, United States

**Keywords:** ovarian cancer, high grade serous ovarian cancer (HGSOC), tumor-infiltrating lymphocytes (TIL), tumor microenvironment (TME), T-cell phenotype

## Abstract

**Introduction:**

Despite predicted efficacy, immunotherapy in epithelial ovarian cancer (EOC) has limited clinical benefit and the prognosis of patients remains poor. There is thus a strong need for better identifying local immune dynamics and immune-suppressive pathways limiting T-cell mediated anti-tumor immunity.

**Methods:**

In this observational study we analyzed by immunohistochemistry, gene expression profiling and flow cytometry the antigenic landscape and immune composition of 48 EOC specimens, with a focus on tumor-infiltrating lymphocytes (TILs).

**Results:**

Activated T cells showing features of partial exhaustion with a CD137^+^CD39^+^PD-1^+^TIM-3^+^CD45RA^-^CD62L^-^CD95^+^ surface profile were exclusively present in EOC specimens but not in corresponding peripheral blood or ascitic fluid, indicating that the tumor microenvironment might sustain this peculiar phenotype. Interestingly, while neoplastic cells expressed several tumor-associated antigens possibly able to stimulate tumor-specific TILs, macrophages provided both co-stimulatory and inhibitory signals and were more abundant in TILs-enriched specimens harboring the CD137^+^CD39^+^PD-1^+^TIM-3^+^CD45RA^-^CD62L^-^CD95^+^ signature.

**Conclusion:**

These data demonstrate that EOC is enriched in CD137^+^CD39^+^PD-1^+^TIM-3^+^CD45RA^-^CD62L^-^CD95^+^ T lymphocytes, a phenotype possibly modulated by antigen recognition on neoplastic cells and by a combination of inhibitory and co-stimulatory signals largely provided by infiltrating myeloid cells. Furthermore, we have identified immunosuppressive pathways potentially hampering local immunity which might be targeted by immunotherapeutic approaches.

## Introduction

Ovarian cancer accounts for 2.5% of all female tumors. Despite its relatively low incidence, it represents the 8^th^ most common cause of death from cancer in women (1). The prognosis of patients affected by epithelial ovarian cancer (EOC) has not substantially changed over the last decades, after platinum-based chemotherapy was introduced, and the majority of patients initially responding to chemotherapy will eventually develop chemoresistance and relapse (2). There is thus an urgent need for innovative and effective therapies.

Despite being considered a “cold tumor” with a low tumor mutational burden, a positive correlation between the amount of tumor-infiltrating lymphocytes (TILs), in particular CD8^+^ cells, and favorable clinical outcome has been reported in EOC, suggesting that this disease might be sensitive to immunotherapeutic approaches, including adoptive T cell therapy (3–10). The analysis of the pattern of the immune infiltration in EOC has revealed that TILs are mainly distributed within the stroma (S-TIL) or both in the stroma and in the epithelium (ES-TIL), with high intra-patient variation. In a minority of cases, EOC is completely devoid of lymphocytes (N-TIL) (11–14). Noticeably, the clinical significance of the infiltration pattern and spatial location of TILs remains poorly investigated (12, 14).

The functional phenotype of TILs has been largely studied in so called “hot tumors” (15), such as melanoma and colorectal cancer (16). Prolonged exposure of tumor-specific T cells to tumor antigens in an immunosuppressive microenvironment often induces an exhausted status in TILs (17), characterized by the co-expression of multiple inhibitory receptors (IRs) and associated to cancer immune evasion. According to these observations, in selected tumors, specific cell-surface signatures in TILs have been identified and proved enriched in tumor-reactive T cells (18–22). Differently from other solid tumors, EOC harbors a low frequency of tumor-specific TILs (23). The identification of a cell surface signature associated with tumor-reactive T cells in EOC is thus more cumbersome and first results are controversial: while none of the markers evaluated by Scheper et al. segregated tumor-specific TILs from bystander lymphocytes (23), other authors found that the expression of CD137 or of PD-1 and the co-expression of CD39 and CD103 in intraepithelial TILs is associated with a high degree of anti-tumor reactivity (19, 24–26). These data depict a complex scenario for EOC, where several key molecules could be involved in the regulation of local immunity. Accordingly, therapy with single immune checkpoint blocking agents inhibiting the PD-1/PD-L1 pathway, alone or in combination with chemotherapy, had limited clinical efficacy (27–29). Furthermore, T-cell exhaustion is largely dependent on the interaction between TILs, cancer cells and myeloid cells. Tumor associated macrophages, for instance, can modulate TILs function through soluble immunosuppressive factors secreted in the tumor microenvironment (17) and by providing costimulatory or inhibitory signals (30–32). In human ovarian cancer, myeloid cells have a dual role: while they favor immunosuppressive microenvironment by TGFβ production, regulatory T cells recruitment and PD-L1 expression (33–35), these cells also provide CD28 costimulation, which is required for reinvigoration of CD8^+^ TILs and for their response to PD-1 blockade, and are the main source of CXCL9, which is essential for T-cell recruitment (36, 37). A more comprehensive characterization of TIL phenotype appears therefore essential to precisely identify the pathways involved in the local suppression of anti-tumor immunity, thus prompting the design of disease-tailored immunotherapeutic regimens, and potentially cell-based immunotherapeutic approaches.

EOCs is characterized by a low mutational burden, that usually associates with a low number of immunogenic neoantigens (38); thus the expression of tumor-associated antigens (TAAs) may better describe the antigenic landscape in EOC patients. New York-esophageal squamous cell carcinoma-1 (NY-ESO-1) antigen has been extensively studied because serum antibodies and specific TILs can be detected in ovarian cancer patients (39). The possibility of generating NY-ESO-1 specific immune responses has been tested in clinical trials, with generation of CD4^+^ and CD8^+^ antigen-specific T-cell responses (40, 41). Cancer Antigen 125 (CA125) and Mucin-1 (MUC-1) are expressed in EOC and have been a target for adoptive immunotherapeutic approaches (42–44). Wilms tumor-1 antigen (WT-1) has emerged as an ideal target for immunotherapy, as it is selectively overexpressed in ovarian carcinoma and its expression positively correlates with survival (45–47).

To unravel the cellular and molecular players active in the EOC microenvironment, we performed an extensive characterization of IRs and IR ligands in EOC immune infiltrate, and we combined this analysis with an evaluation of TAAs expression in neoplastic cells. Our data show that EOC is infiltrated by effector memory T (T_EM_) cells displaying features of activation and partial exhaustion, a phenotype possibly modulated by antigen recognition on neoplastic cells and by a combination of inhibitory and co-stimulatory signals largely provided by infiltrating myeloid cells.

## Methods

### Patients’ selection and samples collection

Forty-eight patients diagnosed with EOC at the Department of Gynecology and Obstetrics, Ospedale San Raffaele (OSR) and undergoing cytoreductive surgery from March 1999 to July 2019 were enrolled in the study. Inclusion criteria were: women older than 18 years, diagnosed with advanced EOC who gave their informed consent for participating to this study. Formalin-fixed and paraffin-embedded (FFPE) tumor specimens were analyzed for all the patients, and for a subgroup of 19 patients also fresh neoplastic tissue, peripheral blood, and ascitic fluid (n=14, depending on the presence of peritoneal ascites) were available. Local ethics committee approved the study.

The following clinicopathological characteristics were registered in a dedicated database: age, histopathological diagnosis, International Federation of Gynecology and Obstetrics (FIGO) stage, breast cancer susceptibility genes (BRCA)1/2 status (either somatic or germline), presence of somatic pathological variants in 21 genes implicated in homology recombination repair (HRR) pathway (48, 49) (**Supplementary Table 1**), type of surgical procedure performed, residual tumor, type of cytoreduction (primary vs interval debulking surgery) and type of chemotherapy and maintenance therapy administered.

Tumors were staged according to FIGO staging system of 2014 (50). Reclassification of cases diagnosed prior to this new staging system was applied retrospectively evaluating surgical reports.

A summary of the clinicopathological characteristics of the included patients is provided in **Supplementary Table 2**.

### Samples’ processing for flow cytometry

Peripheral blood, tumor tissue and when available ascitic fluid were collected at diagnostic surgery, before any chemotherapy treatment. After collection, samples were assigned an anonymized reference by the Institutional Biobanking Service. Tumor specimens were in part paraffine-embedded for IHC, IF and gene expression analyses, and in part stored in Tissue Storage Solution (Miltenyi Biotec) for flow cytometry. All samples were kept at +4°C and processed within 24 hours.

Peripheral blood mononuclear cells (PBMC) were obtained from whole blood by density gradient centrifugation (Lymphoprep, Sentinel diagnostics).

Tissue samples were minced and then digested with the Tumor Dissociation kit in gentleMACS Dissociator (Miltenyi Biotec) according to manufacturer’s instructions. After digestion, samples were filtered with a 40 μm cell strainer (Falcon, Corning) and washed with RPMI-1640 medium (BioWhittaker), then red blood cells were lysed 10 minutes at +4°C with ammonium-chloride-potassium (ACK) lysis buffer (BioWhittaker) and samples were washed again with RPMI-1640 containing 10% Fetal Bovine Serum (FBS), 1% penicillin/streptomycin and 1% L-glutamine (all from BioWhittaker).

Ascitic fluids were centrifuged and treated with ACK buffer as above described.

### Flow cytometry

Freshly processed single-cell suspensions from tissue, blood, and ascites were stained with antibodies listed in **Supplementary Table 3** in the presence of Brilliant Stain Buffer (BD Biosciences). Before incubation with antibodies, dead cells were stained with Zombie Green Fixable Viability Kit (Biolegend) and Fc receptors were blocked with human FcR Blocking Reagent (Miltenyi Biotec) according to manufacturers’ instructions. After staining and washing, samples were fixed with Fixation Buffer (Biolegend), stored at +4°C and acquired within 24 hours. The acquisition of the samples was carried out with LSRFortessa and FACSymphony cytometers (BD Biosciences). Data were analyzed with FlowJo v10 software (BD).

### High-dimensional analysis of flow cytometry data

Only samples acquired with LSRFortessa cytometer were used for high-dimensional analysis. After compensation optimization, events were isolated from the raw .fcs files according to physical parameters (FSC-A, FSC-H and SSC-A), negativity for the viability dye and positivity for the CD3 marker. All live T lymphocytes were then exported to a new .fcs file. Using the application cytoChain (51) data were then optimized for acquisition stability, all the channels’ fluorescence intensities were transformed by the arcSinh function (cofactor 150) and random downsampling to 5000 events per sample was performed. CytoChain was also used for subsequent data handling after concatenation of the optimized flowset as previously described (52). The concatenated flowset was also analyzed by FlowJo v10 (BD) to validate cytoChain results.

After a first t-SNE mapping, CTLA-4 marker showed a low and almost uniform expression, with very little differences among the clusters. Therefore, we decided to remove CTLA-4 from mapping and clustering analysis to exclude a potential confounding effect.

### Immunohistochemistry

Immunohistochemistry (IHC) analyses were performed on FFPE tumor specimens with antibodies listed in **Supplementary Table 4**, and two independent pathologists evaluated the specimens. A triple immunohistochemical staining (CD3, CD20, CD163) was performed to establish the presence and spatial distribution of T (CD3^+^), B (CD20^+^) cells and macrophages (CD163^+^) in the tumor microenvironment (TME). TILs percentage was evaluated in the stroma and in the epithelial tumor cells (14). Consequently, cases were classified by the TILs distribution in epithelial TILs (E-TILs) (higher TILs percentage in the epithelial tumor component than in stroma), stromal TILs (S-TILs) (higher TILs percentage in the stroma than in epithelial tumor component), epithelial and stromal TILs (ES-TILs) (same percentage in both) and no TILs (N-TILs) (TILs absent or less than 1% in the tumor). Furthermore, the expression of IRs (PD-1, LAG-3 and TIM-3) by immune cells or of PD-L1 by both neoplastic and immune cells present in the TME were evaluated. For each marker, the number of positive immune or tumor cells per 100 neoplastic cells was calculated. A panel of TAAs (WT-1, NY-ESO-1, CA125, MUC-1) was also assessed and the percentage of their expression in tumor cells was calculated.

### Gene expression analysis

RNA was extracted from FFPE samples by using Maxwell RSC RNA FFPE Kit and Maxwell RSC Instrument (Promega), according to the manufacturer’s instructions.

Elution was performed in 50 μl and RNA was quantified using the Qubit RNA HS Assay Kit on Qubit 3.0 Fluorometer (ThermoFisher Scientific).

Gene expression analysis was performed using nCounter Prep Station and nCounter Digital Analyzer (NanoString), following the instrument protocol. The nCounter PanCancer IO 360™ Panel (NanoString) was used, with the addition of a custom panel targeting 29 genes (**Supplementary Table 5**). Cartridges were run using the 280 FOV protocol.

After quality control and background correction, gene expression was normalized using the target to housekeeping gene expression ratio with the Advanced Analysis module of the nSolver Analysis Software version 4.0 (NanoString Technologies). Samples were categorized using the combination of Epithelial and Stromal TILs data: TIL-Low (TIL ≤ 8%, n = 24 subjects) and TIL-High (TIL > 8%, n = 22 subjects) groups.

### Immunofluorescence analysis for CD137/CD39

An immunofluorescence (IF) double stain was performed to evaluate the co-expression of CD137 and CD39 in lymphocytes (see **Supplementary Table 6**). CD137^+^CD39^+^ cells were identified in five epithelial intra-tumoral high power field areas (HPF) and five stromal intra-tumoral HPF. Each HPF (40x magnification=0.237575938 mm² area) was selected considering the areas more enriched by CD137^+^CD39^+^ cells.

### Statistics

Statistical analyses were carried out using GraphPad Prism 9 and SPSS version 17.0 softwares. In the presence of two variables, the comparison between groups was carried out using two-way ANOVA followed by Sidak’s multiple comparison test. Comparison between two groups was performed by Wilcoxon (for paired groups) or Mann-Withney (unpaired) tests. Correlation analysis was performed by simple linear regression. Statistical significance was defined as p value <0.05.

Receiver operating characteristic (ROC) analysis was used to identify the levels of CD137^+^CD39^+^ among CD4^+^ and CD8^+^ T lymphocytes and of CD33^+^ among total CD45^+^ cells characterized by the best sensitivity and specificity for the prediction of the presence of CD137^+^CD39^+^PD-1^+^TIM-3^+^CD45RA^-^CD62L^-^CD95^+^ CD4^+^ and CD8^+^ T cells in the same EOC samples.

Survival curves were calculated using the Kaplan-Meier method and were compared with the Log-rank test to assess the statistical significance. Patients were censored when lost to follow up. Cox’s regression model was used to analyze the role of clinicopathological factors as prognostic factors for survival.

NanoString nCounter analysis was based on multivariate linear regression with Benjamini-Hochberg (BH) adjustment. We defined genes as differentially expressed if they displayed an adjusted p value less than 0.1 in a pairwise comparison. Finally, we compared the pathway activity scores with two-tailed Student’s t-test and unpaired Student’s t-test, two-way and an adjusted p value (BH correction) less than 0.05 was set as cut-off.

### Data availability

The datasets generated for this study are available on request to the corresponding authors.

## Results

### Patients’ characteristics

In this study we performed a multidimensional investigation of the tumor microenvironment and antigenic landscape of EOC on biological samples harvested from 48 patients at diagnosis. Patients’ clinicopathological characteristics are summarized in **Table 1** and detailed in **Supplementary Table 2**. Most of the patients (93.7%) presented with advanced-stage disease (stage III and IV), which was high-grade serous ovarian cancer (HGSOC) in 85.4% of cases, thus representing the typical population of patients affected by EOC at diagnosis (53). After surgery, all patients received platinum-based chemotherapy, except for one patient who died soon after diagnostic surgery due to myocardial infarction. First line chemotherapy was followed by maintenance therapy with either bevacizumab (n=13) and/or poly (ADP-ribose) polymerase (PARP)-inhibitor (n=1). PARP-inhibitor was administered as maintenance after platinum-based chemotherapy at subsequent relapse in 9 patients.

**Table 1:** Patients’ characteristics.

Patients (n=48)	
Age (years) – mean ± SD (range)	61.3 ± 11.5 (32-82)
Stage, Grade – n (%)	
IIB G3	2 (4.2)
IIIA G3	1 (2.1)
IIIB G3	2 (4.2)
IIIC G3	31(64.6)
IV G3	12 (25.0)
Histotype – n (%)	
HGSOC	41 (85.4)
EOvC	4 (8.3)
OCCC	3 (6.3)
BRCA mutation – n (%)	
Yes	11 (22.9)
No	37 (77.1)
Survival data - median (range)	
PFS (months) – median (range)	9.0 (0-48)
OS (months) - median (range)	34.0 (4-260)

BRCA, Breast Cancer susceptibility genes; EOvC, Endometrioid Ovarian Cancer; HGSOC, High Grade Serous Ovarian Cancer; OCCC, Ovarian Clear Cell Carcinoma; OS, Overall Survival; PFS, Progression Free Survival.

Twelve patients (25%) underwent a diagnostic biopsy due to widespread disease deemed unresectable at primary surgery and were therefore addressed to neoadjuvant chemotherapy followed by interval debulking surgery. The remaining patients underwent primary cytoreductive surgery. Eleven patients (22.9%) harbored either somatic or germline BRCA pathological variants. Among 29 specimens evaluated, 18 harbored pathological alterations in HRR pathway. Survival data were available for 42 patients. In April 2022, median progression free survival (PFS) was 9.0 ± 2.16 (95%CI 4.76-13.20) and median overall survival (OS) was 34.0 ± 3.34 (95%CI 27.44-40.55) months.

### EOC is infiltrated by TILs with dominant stromal localization and expresses several TAAs

**Figure 1:**
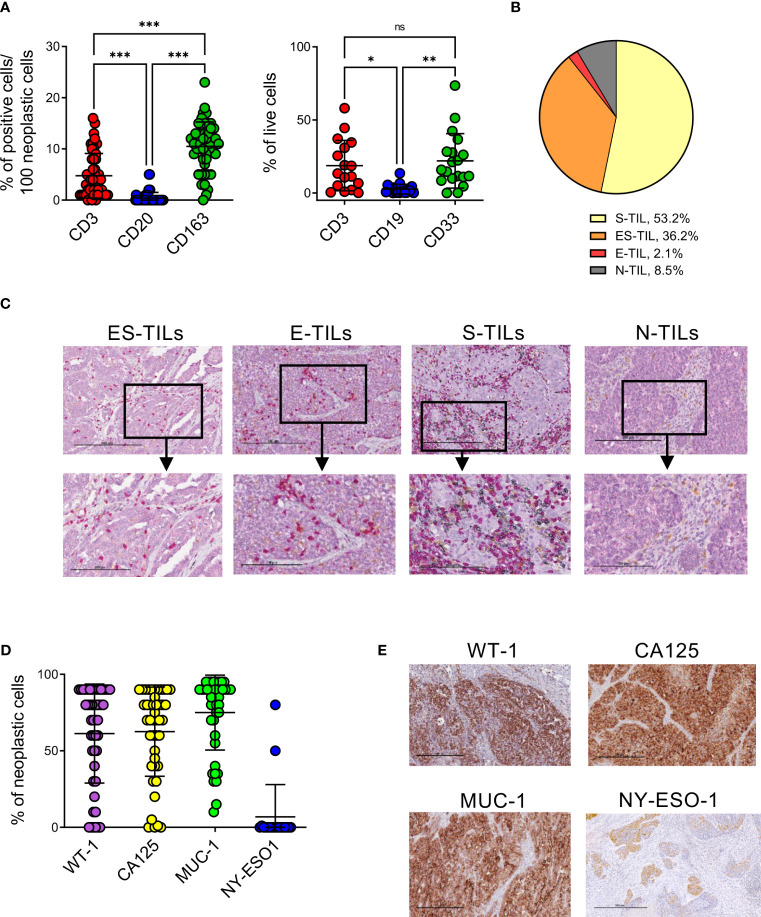
EOC samples are highly infiltrated by T and myeloid cells and express different TAAs. **(A)**, quantification of T and B lymphocytes and myeloid cells infiltrating EOC by IHC (n=47, left panel) and flow cytometry (n=19, right panel). Comparisons by two-way ANOVA: *, p<0.05; **, p<0.01; ***, p<0.001; ns, not significant. **(B)**, distribution of lymphocytes (CD3^+^ or CD20^+^ cells) in n=47 EOC specimens accordingly to their main localization in stroma (S-TIL), epithelium (E-TIL), in both stromal and epithelial areas (ES-TIL) or to their absence (N-TIL, TILs ≤ 1% in all the examined areas). **(C)**, representative staining for ES-TILs, E-TILs, S-TILs and N-TILs by x10 (upper panels) and x20 (lower panels) magnification. **(D)**, expression of the indicated TAAs by neoplastic cells evaluated by IHC (n=47 for WT-1, n=44 for CA125, n=43 for MUC-1, n=19 for NY-ESO). **(E)**, representative staining for the indicated TAAs, x10 magnification.

The type and spatial distribution of the immune infiltrate in EOC was assessed by IHC upon staining for CD3, CD20 and CD163 on the whole cohort, and by flow cytometry by CD3, CD19 and CD33 on 19 freshly processed tumor samples. According to previous data (54), the two techniques were highly concordant in revealing a strong infiltration by T lymphocytes and myeloid cells, with very low levels of B lymphocytes (**Figure 1A**). IHC showed that TILs display a stromal distribution in the majority of patients, with 36% of cases with TIL accumulating both in stromal and epithelial areas (**Figures 1B, C**, **Supplementary Figure 1A**).

To investigate the antigenic landscape of EOC, and identify potential targets for immunotherapy, the expression by cancer cells of WT-1, CA125, MUC-1 and NY-ESO-1 immunogenic TAAs was evaluated by IHC. WT-1, CA125, MUC-1 were expressed by the majority of analyzed samples, with average percentages of TAA expressing cells of 80% (range 0-90, 68% of positive specimens) for WT-1, 70% (range 0-90, 70% of positive specimens) for CA125 and 85% (range 15-95, 81% of positive specimens) for MUC-1. NY-ESO-1 showed the lowest expression levels in all analyzed samples (median 0, range 0-80, 5% of positive specimens) (**Figures 1D, E**).

No differences were observed in the immune composition or TAAs expression of the specimens with Ovarian Clear Cell Carcinoma (OCCC) or Endometrioid Ovarian Cancer (EOvC) histology compared to HGSOC (**Supplementary Figures 2A, B**).

### The gene expression signature of highly TIL-infiltrated EOCs indicate an active T cell-myeloid cross-talk

**Figure 2:**
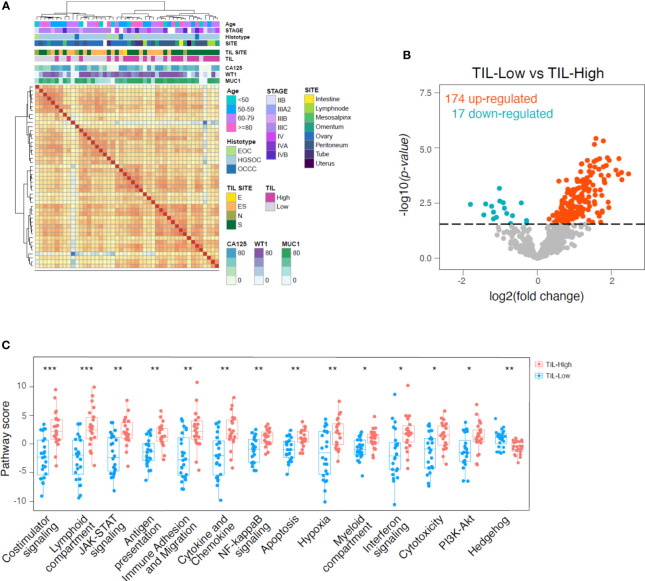
Higher expression of cytotoxicity, anti-tumor chemokines and myeloid-related genes in TIL-High samples. **(A)**, heatmap showing the correlation between 46 surgical specimens according to gene expression profile. Unsupervised clustering using maximum distance and ward.D2 linkage. Annotations of the samples on the top of the heatmap indicate age, histopathological and biological features according to the color legends. **(B)**, volcano plot showing differentially regulated genes derived from NanoString analysis of 46 surgical specimens separated by TIL percentage into TIL-Low (TIL<8% of tumor cells, n=24) and TIL-High (TIL>8% of tumor cells, n=22) groups. Eight percent of TIL content has been used as threshold being the median percentage of TILs infiltration in the evaluated samples. Blue and red dots indicate genes significantly upregulated in TIL-Low and TIL-High, respectively (adjusted p value < 0.1, BH correction). **(C)**, boxplots showing pathways upregulated or downregulated in TIL-High group compared to TIL-Low group. Pathway scores are calculated as the first principal component of the pathway genes’ normalized expression. Statistical significance by two-tailed Student’s t-test and BH correction: *, p<0.05; **, p<0.01; ***, p<0.001.

The gene expression profiles of our EOC samples were evaluated with a panel of 799 immune and cancer-related genes by the NanoString platform. The correlation matrix identified a good clusterization between samples identified by TIL content above or below the median value (TIL-High and TIL-Low, respectively), while other clinical or biological parameters had lower impact on the molecular signature (**Figure 2A**). We performed pairwise comparison, obtaining 174 and 17 deregulated genes (p<0.1, Benjamini-Hochberg correction, BH) specifically high expressed in TIL-High and TIL-Low tumors, respectively (**Figure 2B**, **Supplementary Table 7**). By performing pathway enrichment analysis (**Figures 2B, C**, **Supplementary Figure 1B**), in TIL-High samples we observed increased expression of genes related to T-cell recruitment and accumulation (e.g. CD3E, CD8A, CD4, CCL5, CXCR6, CXCL9, CXCL13, IL2RB, IL2RG, CD2, TRAT1, ITGAL and CCL18), lymphocyte activation and costimulation (LCK, ITGAL, CD48, STAT4, CD96) and antigen processing and presentation (HLA-DRB1, CD74, CD96, CTSS, PSMB9, HLA-DPA1) (36, 55–58). Moreover, also genes typical of myeloid cells (CD33, CXCL9, CD48, APOE, CD74, HLA-DRB1, CD96, IRF4, HCK) and genes induced by interferons (CXCL9, CD48, GBP4, CCL5, HLA-DRB1, LAG3, NLRC5, CD69, IRF2, IRF4, PSMB9) (36, 59–61) were expressed. Importantly, in TIL-High samples we also detected an upregulation of genes associated with a better prognosis of ovarian cancer patients (CD8A, CD4, LCK, CCL5, CXCR6, CXCL9, CXCL13, CD27, CD48RO, APOE, GBP4, TRAT1) (3, 36, 56–58, 62–69). Interestingly, in TIL-High samples we observed a downregulation of genes belonging to the Hedgehog pathway (**Figure 2C**, **Supplementary Figure 1B**, **Supplementary Table 7**), which is associated with EOC progression, *in vitro* chemoresistance and reduced response to anti-PD-L1 therapy in mouse models (70–72).

Overall, these data revealed a distinctive microenvironment in TILs-enriched tumor samples characterized also by myeloid cell enrichment and activation, and suggestive of a crosstalk between innate and adaptive immune cells.

### T lymphocytes co-expressing activation and exhaustion markers are enriched in EOC

**Figure 3:**
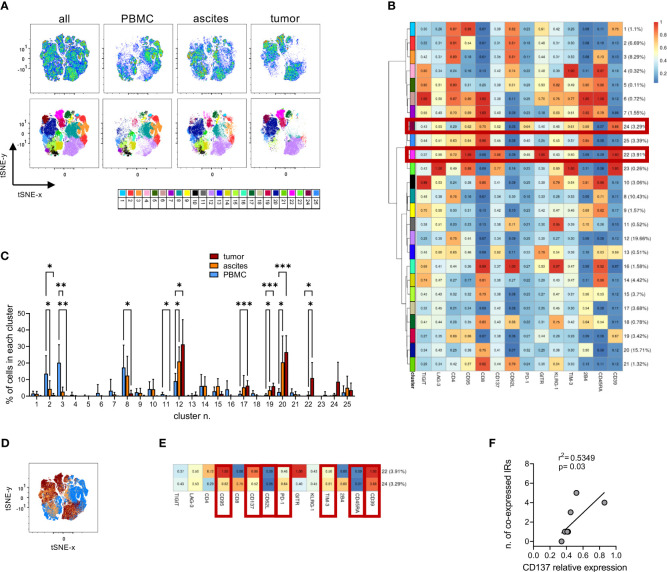
High-dimensional analysis of T cells identifies different T-cell subsets in PBMCs, ascitic fluids and tumors. **(A)**, tSNE analysis (upper panels) and Flow-SOM metaclusters overlay (lower panels) of CD3^+^ T cells from either all concatenated samples or PBMC, ascites and tumor specimens from 8 high-grade EOC patients. **(B)**, heatmap for the 25 Flow-SOM metaclusters; the ratio of fluorescence intensity with respect to the maximum is reported. Brown boxes highlight the clusters expressed only in tumor specimens. **(C)**, metaclusters frequency in T cells from matched PBMC (light blue), ascites (orange) and tumor (brown) specimens. Analysis by two-way Anova with Sidak’s multiple comparison: *, p<0.05; **, p<0.01; ***, p<0.001. **(D)**, metaclusters overlay for all concatenated samples showing the differential contribution for each type of tissue as in C. **(E)**, detail of metaclusters n. 22 and 24. Brown boxes indicate the markers enriched or reduced in both clusters. **(F)**, correlation by linear regression analysis between the relative expression of CD137 in each mc and the number of IRs co-expressed in the same mc. Only metaclusters with >1% of cells in concatenated neoplastic samples have been analyzed. IRs were considered positive when their relative expression was >0.5.

To reveal the activation, exhaustion and differentiation status of TILs, we first performed IHC staining for PD-1, LAG-3 and TIM-3, which revealed strong expression of these IRs by lymphoid cells within EOC (**Supplementary Figure 3**). Considering the lack of B cells, T lymphocytes or NK cells might be the major lymphoid subsets expressing those IRs in our specimens. To gain more insights on the level of T-cell exhaustion, in selected cases, we comparatively analyzed TILs, matched T lymphocytes accumulating in the ascitic fluid and circulating T cells by polychromatic flow cytometry. To this aim we designed a flow cytometry panel able to simultaneously identify activated and exhausted T cells within each T-cell memory subset. We examined freshly processed matched PBMC, ascites and tumor samples from 8 high-grade EOC patients. High-dimensional analysis performed using the application cytoChain (51) allowed to identify 25 site-specific phenotypic T-lymphocyte metaclusters (mc), with major differences observed between PBMC and tumor and an intermediate pattern in T cells from ascitic fluid (**Figures 3A, B**). Metacluster (mc)2 and mc3 were uniquely expressed in peripheral blood (mc2 p=0.03 for PBMC vs ascites and p=0.04 for PBMC vs tumor, mc3 p=0.002 for PBMC vs ascites and p=0.003 for PBMC vs tumor) and included CD4^+^ T lymphocytes with naïve (T_N_, CD45RA^+^CD62L^+^CD95^-^, mc3) and central memory (T_CM_, CD45RA^-^CD62L^+^CD95^+^, mc2) phenotype, while mc8 was expressed in both PBMC and ascitic compartments (p=0.03 for PBMC vs tumor) and was composed by late-stage (T_EMRA_, CD45RA^+^CD62L^-^) CD4^+^ T cells. Conversely, mc12, mc17, mc19 and mc20 were enriched in both neoplastic and ascitic samples compared to PBMC (mc12 p=0.01 for PBMC vs both ascites and tumor, mc17 p<0.001 for PBMC vs tumor, mc19 p=0.02 for PBMC vs ascites and p<0.001 for PBMC vs tumor, mc20 p=0.03 for PBMC vs ascites and p<0.001 for PBMC vs tumor) and consisted of CD4^+^ or CD8^+^ effector memory cells (T_EM_, CD45RA^-^CD62L^-^, mc12, mc19 and mc20) or of double-negative lymphocytes (mc17). Exhaustion markers were found only at low levels in these mc (GITR in mc2 and mc3, TIGIT in mc8, 2B4 in mc17 and mc20), with the only exception of discrete levels of CD39 in mc19. Importantly, no expression of CD137, a marker of recently activated T cells (73), or co-expression of multiple IRs has been found in these metaclusters (**Figures 3B–D**).

Interestingly, CD137 expression was restricted to mc22 and mc24, which were present only in TILs but not in T lymphocytes from PBMC or ascites (mc22 p=0.04 for tumor vs both ascites and PBMC, **Figure 3C**). These metaclusters consisted of either CD4^+^ (mc22) or CD8^+^ (mc24) T cells sharing a T_EM_ phenotype, high CD39 and CD95 expression and higher expression of PD-1 and TIM-3 compared to the overall T cell population, identifying a CD137^+^CD39^+^PD-1^+^TIM-3^+^CD45RA^-^CD62L^-^CD95^+^ signature for T lymphocytes exclusively present in the tumors (**Figures 3B, E**). Within mc expressed in TILs, CD137 relative expression levels correlated with the number of positive IRs in the same mc (**Figure 3F**), underlining the contemporary presence of activation and exhaustion markers. We then dissected the expression of the identified mc in TILs from the 8 neoplastic samples used for high-dimensional analysis, which derived from tumors with different clinical and biological features. Mc24 was enriched (p<0.001) in lesions characterized by mutations in genes belonging to HRR pathway and in samples with an ES-TIL infiltration pattern (p=0.02 for S-TIL vs ES-TIL, p<0.001 for N-TIL vs ES-TIL). Mc22 was enriched in samples with high TIL accumulation compared to those without lymphocyte infiltration (p=0.02 for S-TIL vs N-TIL) and, most importantly, in tumors from patients with PFS longer than the median of the whole cohort (p=0.01). Both mc22 and mc24 display a trend for enrichment in tumors expressing WT-1 (**Supplementary Figure 4**). Interestingly, metacluster 12, representing resting non-exhausted CD4^+^ T_EM_ cells, decreased in samples with mutations in HRR pathway (p<0.001), high WT-1 expression (p=0.02) and in tumors from long-surviving patients (p<0.001). These data suggest a beneficial effect of the tumor infiltration by activated T lymphocytes, even though such cells co-express exhaustion-related molecules.

### Tumor infiltration by CD137^+^CD39^+^ T lymphocytes is associated with better prognosis

**Figure 4:**
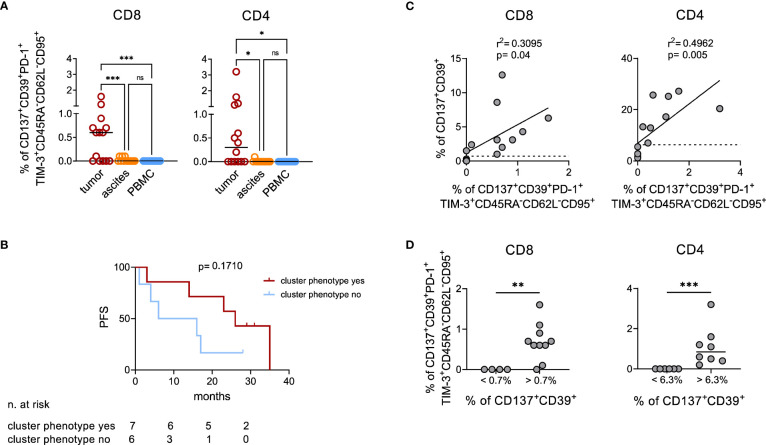
Manual analysis confirms co-expression of activation and exhaustion markers by TILs. **(A)**, quantification by manual gating of CD137^+^CD39^+^PD-1^+^TIM-3^+^CD45RA^-^CD62L^-^CD95^+^ cells among CD8^+^ (left) and CD4^+^ (right) T lymphocytes in matched tumor (n=14), ascites (n=13) and PBMC (n=14) samples from EOC patients. Bars, median values. Analysis by one-way Anova: *, p<0.05; ***, p<0.001; ns, not significant. **(B)**, Kaplan-Meier estimates of PFS in 13 patients with or without detectable CD137^+^CD39^+^PD-1^+^TIM-3^+^CD45RA^-^CD62L^-^CD95^+^ T cells in their tumors (cut-off >0.1% of CD137^+^CD39^+^PD-1^+^TIM-3^+^CD45RA^-^CD62L^-^CD95^+^ cells among either CD8^+^ or CD4^+^ T lymphocytes). Lines indicate censored data. Comparisons by Log-rank test. **(C)**, correlation by linear regression analysis between the percentage of CD137^+^CD39^+^PD-1^+^TIM-3^+^CD45RA^-^CD62L^-^CD95^+^ and of CD137^+^CD39^+^ cells among CD8^+^ (left) and CD4^+^ (right) T lymphocytes from n=14 EOC specimens. Dotted lines indicate the values of CD137^+^CD39^+^ T lymphocytes which identify with the best sensitivity and specificity by ROC analysis the presence of CD137^+^CD39^+^PD-1^+^TIM-3^+^CD45RA^-^CD62L^-^CD95^+^ cells (1.8% for CD8^+^ T cells and 6.3% for CD4^+^ T cells). **(D)**, frequency of CD137^+^CD39^+^PD-1^+^TIM-3^+^CD45RA^-^CD62L^-^CD95^+^ among CD8^+^ (left) and CD4^+^ (right) T cells from n=14 EOC specimens containing percentages of CD137^+^CD39^+^ cells below or over the thresholds identified with ROC analysis. Analysis by Mann-Whitney test: **, p<0.01; ***, p<0.001.

Manual gating cytofluorimetric analysis on the freshly processed samples investigated above and on additional samples from 6 EOC patients (**Supplementary Figure 5**) confirmed the presence in neoplastic lesions of both CD8^+^ and CD4^+^ T lymphocytes with the CD137^+^CD39^+^PD-1^+^TIM-3^+^CD45RA^-^CD62L^-^CD95^+^ phenotypic signature previously identified by high dimensional analysis. This cellular subpopulation was not detectable in T cells from ascitic fluid nor in peripheral blood of the same patients (**Figure 4A**; CD8^+^: p<0.001 for tumor vs both PBMC and ascites, CD4^+^: p=0.02 for tumor vs ascites and p=0.01 for tumor vs PBMC). Interestingly, also in this small cohort of the 13 patients for which PFS was available, we found a trend for better PFS in patients with detectable CD137^+^CD39^+^PD-1^+^TIM-3^+^CD45RA^-^CD62L^-^CD95^+^ TILs (**Figure 4B**, median PFS 26 vs 11 months, hazard ratio 0.45, 95%CI of ratio 0.12 to 1.71). We thus re-analyzed the data in search of a combination of few cell surface markers correlated with this complex signature, to be exploited in a larger cohort of patients. Interestingly, we observed that the percentage of CD137^+^CD39^+^ T cells within EOC samples positively correlates with the CD137^+^CD39^+^PD-1^+^TIM-3^+^CD45RA^-^CD62L^-^CD95^+^ signature, in both CD4^+^ and in CD8^+^ T cells (**Figure 4C**). ROC analysis identified the values of 1.8% CD137^+^CD39^+^/CD8^+^ (area under the curve (AUC)=0.978, p=0.004) and 6.3% CD137^+^CD39^+^/CD4^+^ (AUC=1, p=0.002) as the best thresholds for the presence of CD137^+^CD39^+^PD-1^+^TIM-3^+^CD45RA^-^CD62L^-^CD95^+^ CD8^+^ or CD4^+^ T cells, respectively (**Supplementary Table 8**, **Figure 4D**). Furthermore, CD137^+^CD39^+^ T cells showed increased expression of several IRs compared to the total CD8^+^ and CD4^+^ T lymphocytes (**Supplementary Figure 6A**).

**Figure 5:**
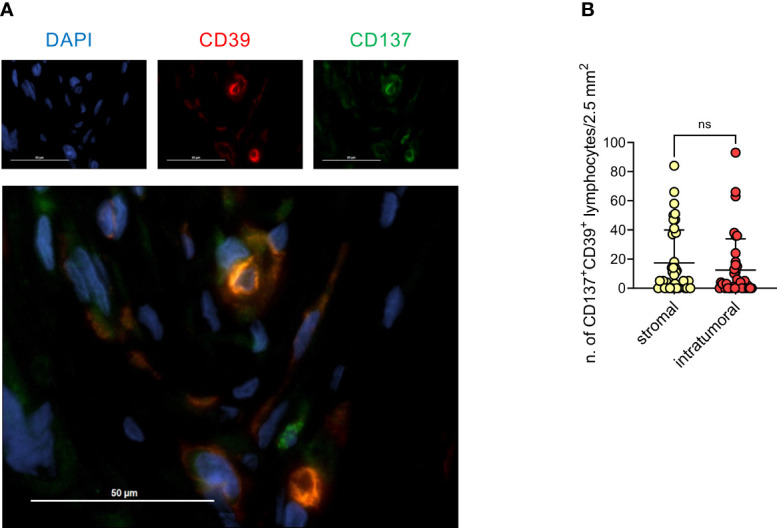
Presence of CD137^+^CD39^+^ lymphoid cells in the stomal compartment is associated with better prognosis. **(A)**, Representative immunofluorescence staining for CD39 (red), CD137 (green) and DAPI (blue), 40x magnification. **(B)**, quantification by immunofluorescence of CD137^+^CD39^+^ lymphoid cells in either the stromal or the epithelial compartment of 38 EOC specimens. Bars indicate mean values ± SD. Analysis by Wilcoxon test: n.s., not significant.

Based on these findings, we screened all available (n=38) EOC specimens by IF for CD137 and CD39 (**Figure 5A**). Double-positive lymphocytes were detected, albeit at low numbers, in both stromal and epithelial compartments (**Figure 5B**). Interestingly, we observed a trend for a better survival for HGSOC patients with more CD137^+^CD39^+^ TILs compared to patients with lower CD137^+^CD39^+^ TILs (stromal TILs: median PFS 13 vs 6.5 months, hazard ratio for disease progression 0.50, 95%CI of ratio 0.23 to 1.09. Intra-epithelial TILs: median PFS 13 vs 6.5 months, hazard ratio for disease progression 0.50, 95%CI of ratio 0.23 to 1.07) (**Supplementary Figure 6B**), while total T-cell infiltration did not correlate with PFS (data not shown).

No major differences among different EOC histotypes were observed for T-cell phenotype, including the presence of CD137^+^CD39^+^PD-1^+^TIM-3^+^CD45RA^-^CD62L^-^CD95^+^ signature or of CD137^+^CD39^+^ lymphocytes (**Supplementary Figures 2C–E**).

Overall, these data show that in EOC a T-cell infiltrate enriched in cells co-expressing CD137 and CD39 has a potential positive prognostic implication.

### Myeloid cells expressing IR ligands are abundant in EOC specimens enriched in CD137^+^CD39^+^PD-1^+^TIM-3^+^CD45RA^-^CD62L^-^CD95^+^ T cells

**Figure 6:**
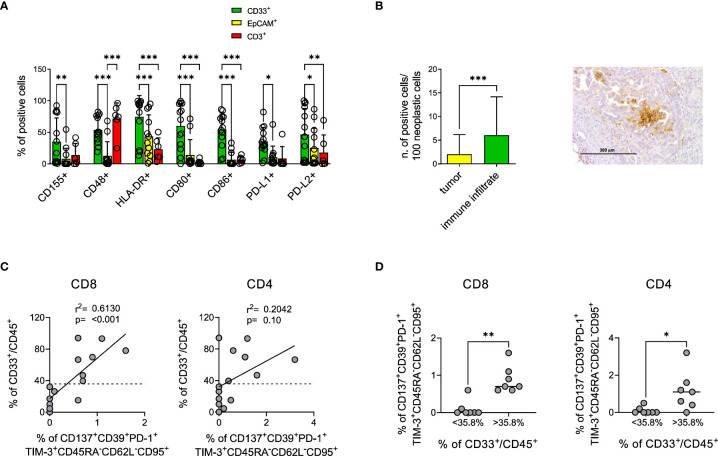
Myeloid cells mainly express IR ligands in the tumor microenvironment and correlate with CD137^+^CD39^+^PD-1^+^TIM-3^+^ T_EM_. **(A)**, percentage of myeloid (green bars, CD33^+^ n=13) epithelial (yellow bars, EpCAM^+^ n=13) or T (red bars, CD3^+^ n=6) cells from EOC samples expressing the indicated IR ligands by flow cytometry analysis. Dots indicate individual values. Analysis by two-way Anova with Sidak’s multiple comparison: *, p<0.05; **, p<0.01; ***, p<0.001. **(B)**, upper panel, expression of PD-L1 by neoplastic or immune cells in n=36 EOC samples by IHC. Analysis by Wilcoxon test; *** p<0.001. Right panel, representative IHC staining for PD-L1 showing predominant expression by myeloid-like cells. **(C)**, upper panels, correlation by linear regression analysis between the percentage of CD137^+^CD39^+^PD-1^+^TIM-3^+^CD45RA^-^CD62L^-^CD95^+^ among CD8^+^ (left) and CD4^+^ (right) T lymphocytes and of CD33^+^ among CD45^+^ cells from n=14 EOC specimens. Dotted lines indicate the value of CD33^+^ cells which identify with the best sensitivity and specificity by ROC analysis the presence of CD137^+^CD39^+^PD-1^+^TIM-3^+^CD45RA^-^CD62L^-^CD95^+^ cells (35.8%). **(D)**, presence of CD137^+^CD39^+^PD-1^+^TIM-3^+^CD45RA^-^CD62L^-^CD95^+^ among CD8^+^ (left) and CD4^+^ (right) T cells from n=14 EOC specimens containing percentages of CD33^+^ cells among CD45^+^ cells below or over the threshold identified with ROC analysis. Analysis by Mann-Whitney test: *, p<0.05; **, p<0.01.

Ligands for IRs might be expressed by cancer cells and by different cellular components of the tumor microenvironment (74–76). To identify the cell type possibly engaging IRs in TILs, we analyzed by flow cytometry the expression of several molecules on neoplastic and myeloid cells and T lymphocytes; B lymphocytes were not characterized due to their low frequency in EOC samples. Our data show that a large fraction of myeloid cells co-expressed IR ligands in the majority of EOC samples, with a prevalence of HLA-DR, CD80, CD86, PD-L1 and CD48. In a lower proportion of samples, we also observed myeloid cells expressing high percentages of PD-L2 or CD155. Conversely, cancer cells, defined by EpCAM expression, scarcely expressed IR ligands, with the exception of HLA-DR and of a minority of samples highly positive for PD-L2. T lymphocytes displayed in almost all the samples high levels of CD48, while CD155, HLA-DR and PD-L2 were more heterogeneous and the other ligands mainly absent on this subset (**Figure 6A**, **Supplementary Figure 7**). IHC confirmed the prevalent expression of PD-L1 by myeloid cells (**Figure 6B**). Although the expression of TIM-3 ligands could not be evaluated on the cells from fresh neoplastic specimens, gene expression analysis showed increased expression of Galectin-9 associated with increased expression of TIM-3 in TIL-High compared to TIL-Low samples. This finding was confirmed by IHC assessment of Galectin-9 expression on immune cells (**Supplementary Figure 8**).

Interestingly, the abundance of myeloid cells in EOC samples positively correlated with the presence of CD8^+^ T lymphocytes characterized by the CD137^+^CD39^+^PD-1^+^TIM-3^+^CD45RA^-^CD62L^-^CD95^+^ signature (**Figure 6C**). Furthermore, ROC analysis identified the value of 35.8% CD33^+^/CD45^+^ as the best threshold for the presence of CD137^+^CD39^+^PD-1^+^TIM-3^+^CD45RA^-^CD62L^-^CD95^+^ in CD8^+^ T cells (AUC=0.933, p=0.009) (**Supplementary Table 9A**, **Figure 6D**). Similar trends were detected for CD4^+^ T cells (AUC=0.688, p=0.25) (**Figures 6C, D**, **Supplementary Table 9B**).

These results, according to gene expression data, indicate the presence in EOC samples of a cooperation between TILs and myeloid cells, the latter providing not only co-stimulation and antigen presentation but also triggering of IRs on T lymphocytes.

## Discussion

Cancer immunotherapy is an innovative therapeutic approach that already proved effective in several tumor types. However, the use of checkpoint inhibitors and/or of engineered cellular products should be tailored to the specific immunosuppressive pathways active in each disease. Here, we analyzed the EOC microenvironment by complementary approaches including multiparametric flow cytometry, gene expression profiling and immunofluorescence to identify specific immunological patterns characterizing this aggressive disease. Results pinpoint to a specific signature composed of effector-memory TILs co-expressing the activation marker CD137 and high levels of immunosuppressive molecules such as CD39, TIM-3 and PD-1, a phenotype suggestive of recent T-cell activation and continuous antigen exposure (73, 77, 78). No or very low expression of the other IRs evaluated, including TIGIT, LAG-3, CTLA-4, GITR, 2B4 and KLRG1, was detected in these cells, suggesting that they are not completely exhausted. The CD137^+^CD39^+^PD-1^+^TIM-3^+^CD45RA^-^CD62L^-^CD95^+^ TILs signature identified by flow cytometry correlated with a simplified CD137^+^CD39^+^ T cell signature, which could be exploited and validated by IF on a wider set of samples. In this cohort, a trend for a better PFS was associated with the presence of CD137^+^CD39^+^ lymphocytes in the stromal compartment of HGSOC, which is more frequently infiltrated by T lymphocytes compared to epithelial areas. Although this correlation might not be completely correct due the heterogeneity in rates of optimal cytoreduction and maintenance treatments, and needs to be validated in larger cohorts of patients receiving homogeneous therapy, these findings possibly suggest that activated CD137^+^ T cells retain functionality, despite the co-expression of multiple inhibitory receptors. In line with this hypothesis, in different studies, CD137 expression on EOC TILs identified functional and tumor-specific lymphocytes among either CD39^+^, CD103^+^ or PD-1^+^ TILs (19, 26), and agonistic CD137-specific antibodies enhanced the effect of anti-PD-1 inhibition on cytokines production by CD39^+^ EOC TILs (79).

CD137^+^CD39^+^PD-1^+^TIM-3^+^CD45RA^-^CD62L^-^CD95^+^ T cells were barely detectable in the peripheral blood or even in the ascitic fluid from the same patients, suggesting that this functional phenotype is dependent by the EOC microenvironment. We observed that, while neoplastic cells expressed several immunogenic TAAs, which might activate TILs, infiltrating myeloid cells dominantly expressed IR ligands, that could sustain the phenotype of TILs.

Furthermore, both gene expression and flow cytometry analyses suggest a strong association between T-cell infiltrate and the presence of myeloid cells providing co-stimulation and antigen-presentation. In agreement, antigen-presenting cells in intraepithelial myeloid cell niches have been described to provide TILs co-stimulation, which was essential for the response to PD-1 blockade *in vitro* in EOC (37).

Analysis on samples with rare histologies (clear cell carcinoma or endometroid), did not show any difference from HGSOC in the composition or localization of the immune infiltrate, although this finding needs to be validated in larger cohorts given the low sample size.

Three TAA (WT-1, MUC-1 and CA125) were found highly expressed by neoplastic EOC cells, suggesting a potential role for TAAs in inducing an anti-tumor response in EOC.

One main limitation of the present study is related to the wide time span during which all the patients have been treated. Over this time period, surgical approaches and therapeutic strategies have changed. In particular, first line maintenance treatment such as bevacizumab and PARP inhibitors were not available at the beginning of the study, and became available only for the patients enrolled later than 2013 for bevacizumab and 2016 for PARPis maintenance. These maintenance therapies have shown to be effective in prolonging PFS and in modulating the tumor immune microenvironment (80–82). It is clear that being these treatments not completely homogeneous and considering the number of analyzed samples, caution is needed when interpreting positive or negative correlations with PFS. Moreover, tumor biopsies at primary surgery were retrieved from different sites (petitoneum, omentum or ovary) and this might affect the presented comparisons. Another limitation is the lack of TMB assessment and correlation to our findings, that will need to be addressed in future (83–86).

Collectively, our results depict an EOC microenvironment where activated antigen-experienced CD137^+^CD39^+^PD-1^+^TIM-3^+^CD45RA^-^CD62L^-^CD95^+^ T lymphocytes and myeloid cells co-exist and possibly cooperate to drive local immune response. These results suggest that the combined inhibition of multiple exhaustion-related pathways might be needed for effective immunotherapy in EOC, and identify CD39, PD-1 and TIM-3 as potential targets for the reinvigoration of anti-tumor immunity.

## Data availability statement

NanoString data generated for this study are available in a public, open access repository (GEO Database) under the accession number GSE243751. Other datasets generated for this study are available on request to the corresponding author.

## Ethics statement

The studies involving humans were approved by Comitato Etico Ospedale San Raffaele Milano. The studies were conducted in accordance with the local legislation and institutional requirements. The participants provided their written informed consent to participate in this study.

## Author contributions

ET designed the study, conducted laboratory experiments, analyzed and interpreted data and wrote the paper; AB designed the study, provided clinical data and samples, analyzed and interpreted data and wrote the paper; JW conducted laboratory experiments and analyzed data; MS’A and GT performed IHC and IF analyses and provided clinical data; EB, MR, and MGC performed, analyzed and interpreted the results of NanoString assay; CBa participated in statistical analyses; AP and FS participated to laboratory experiments; DA and FM participated to high dimensional analysis of flow cytometry data; GMag performed and analyzed the results of BRCA and HRR-pathway mutations; ERu, MCM, FT, MC, and BS participated to data discussion and interpretation; ERa. RC, LB, and GC provided clinical data and samples; CD designed and supervised the study; GM and CBo designed and supervised the study and wrote the paper. All authors contributed to the article and approved the submitted version.
