# Supplementary material for: Epithelial ovarian cancer is infiltrated by activated effector T cells co-expressing CD39, PD-1, TIM-3, CD137 and interacting with cancer cells and myeloid cells
Source: Front Immunol. 2023 Oct 4;14:1212444. doi: 10.3389/fimmu.2023.1212444 (PMC10585363; doi:10.3389/fimmu.2023.1212444)
Supplement: Supplementary file 2 [file Presentation_1.pdf]

SUPPLEMENTARY MATERIAL FOR:

Epithelial Ovarian Cancer is infiltrated by activated effector T cells co-expressing CD39 PD-1, TIM-3, CD137 and interacting with cancer cells and myeloid cells

Elena Tassi[1,2*], Alice Bergamini[3,4*], Jessica Wignall[1], Miriam Sant'Angelo[5], Emanuela Brunetto[5], Chiara Balestrieri[1], Miriam Redegalli[5], Alessia Potenza[1], Danilo Abbati[1], Francesco Manfredi[1,3], Maria Giulia Cangi[5], Gilda Magliacane[5], Fabiola Scalisi[5], Eliana Ruggiero[1], Maria Chiara Maffia[1], Federica Trippitelli[1], Emanuela Rabaiotti[4], Raffaella Cioffi[4], Luca Bocciolone[4], Giorgio Candotti[4], Massimo Candiani[3,4], Gianluca Taccagni[5], Birgit Schultes[6], Claudio Doglioni[3,5], Giorgia Mangili[4] and Chiara Bonini[1,2,3].

[1]Experimental Hematology Unit, Division of Immunology, Transplantation and Infectious Disease, IRCCS Ospedale San Raffaele, Milano, Italy

[2]Cell Therapy Immunomonitoring Laboratory (MITiCi), Division of Immunology, Transplantation and Infectious Diseases, IRCCS Ospedale San Raffaele, Milan, Italy

[3]Università Vita-Salute San Raffaele, Milan, Italy

[4]Department of Obstetrics and Gynecology, IRCCS Ospedale San Raffaele, Milan, Italy

[5]Department of Surgical Pathology, IRCCS Ospedale San Raffaele, Milan, Italy

[6]Intellia Therapeutics, Inc., Cambridge, MA, USA

*These authors contributed equally to this work

Corresponding authors: Chiara Bonini (bonini.chiara@hsr.it)

Giorgia Mangili (mangili.giorgia@hsr.it)

Supplementary Tables

Supplementary Table 1. List of full-length genes included in HRR-pathway panel

ATM	CHEK2	RAD51
BARD1	FANCD2	RAD51B
BRCA1	MRE11	RAD51C
BRCA2	NBN	RAD51D
BRIP1	PALB2	RAD52
CDK12	PARP1	RAD54L
CHEK1	RAD50	TP53

Supplementary Table 2. Patients' clinical characteristics

Patient	Age	Hystology	FIGO Stage Grade	BRCA status	HRR status	Tumor specimen	Diagnosis	RT after primary surgery (cm)	First line treatment	Response to first line	Relapse	PFS1 (months)	Status	OS (months)	Last FUP	Analysis
**1**	51	HGSOC	IIIC G3	gBRCA1	Altered	Primary	21/09/2009	RT< 0.1	Carboplatin + Paclitaxel	CR	YES	15	AWD	124	18/01/2020	IHC, GEP, IF
**2**	39	HGSOC	IIIC G3	WT	Altered	Primary	11/03/1999	0	Carboplatin + Paclitaxel	CR	YES	26	NED	254	22/05/2020	IHC, GEP, IF
**3**	81	HGSOC	IIIC G3	WT		Primary	20/08/2012	>10	Carboplatin+ Paclitaxel	PD	YES	0	DOD	23	04/07/2014	IHC, GEP
**4**	61	HGSOC	IIIC G3	gBRCA1	Altered	primary	24/10/2006	>10	NACT (Carboplatin + Paclitaxel) + IDS	CR	YES	11	DOD	125	01/03/2017	IHC, GEP, IF
**5**	63	HGSOC	IIIC G3	WT		Primary	21/07/2016	>10	NACT (Carboplatin + paclitaxel) + IDS	PR	YES	7	DOD	56	25/03/2021	IHC, GEP
**6**	49	HGSOC	IIIC G3	WT	WT	Primary	30/11/2011	>10	Carboplatin+ Paclitaxel	PR	YES	1	DOD	35	20/10/2014	IHC, IF
**7**	65	HGSOC	IIIC G3	WT		Primary	21/03/2003	>10	NACT (Carboplatin + paclitaxel) + IDS	CR	YES	13	DOD	87	07/07/2010	IHC, GEP, IF
**8**	67	HGSOC	IIIC G3	WT	Altered	Primary	12/02/2015	0	Carboplatin+ Paclitaxel	CR	YES	8	NED	71	23/01/2021	IHC, GEP, IF
**9**	68	HGSOC	IV G3	WT		Primary	01/09/2017	>10	NACT (Carboplatin + paclitaxel) + IDS	PR	YES	2	DOD	23	15/08/2019	IHC, GEP, IF
**10**	62	HGSOC	IIIC G3	WT		Primary	12/08/2015	>10	Carboplatin + Paclitaxel	PR	YES	4	DOD	24	01/09/2017	IHC, IF
**11**	48	HGSOC	IIIC G3	gBRCA2	Altered	Primary	23/07/2014	>10	NACT+ IDS (Carboplatin+ Paclitaxel)	CR	YES	12	DOD	58	21/05/2019	IHC, GEP
**12**	71	HGSOC	IIIC G3	WT		Primary	27/03/2015	<1	Carboplatin	PR	YES	7	DOD	34	26/05/2018	IHC, GEP, IF
**13**	79	HGSOC	IIIC G3	WT		Primary	11/09/2012	>10	Carboplatin	PD	YES	3	DOD	11	01/09/2013	GEP
**14**	64	HGSOC	IIIC G3	WT		Primary	08/06/2014	>10	NACT+ IDS (Carboplatin + paclitaxel)	CR	YES	9	DOD	32	28/02/2017	IHC, GEP
**15**	60	HGSOC	IIIC G3	WT		Primary	21/02/2016	0	Carboplatin + Paclitaxel + Bevacizumab	PR	YES	5	DOD	11	31/01/2017	IHC, GEP, IF
**16**	58	HGSOC	IIIC G3	sBRCA2	Altered	Primary	28/02/2006	0	Carboplatin + Paclitaxel	CR	YES	40	DOD	134	01/05/2017	IHC, GEP, IF
**17**	52	HGSOC	IV G3	WT		Primary	06/10/2006	5	NACT+ IDS (Carboplatin + paclitaxel)	CR	YES	11	DOD	18	30/04/2008	IHC, GEP, IF
**18**	68	EOvC	IV G3	WT	Altered	Primary	18/01/2012	>1	Carboplatin + Paclitaxel	PR	YES	2	DOD	13	01/03/2013	IHC, GEP, IF
**19**	66	HGSOC	IIIC G3	WT		Primary	30/11/2006	>10	NACT +IDS (Carboplatin + Paclitaxel)	PR	YES	4	DOD	29	01/05/2009	IHC, GEP, IF
**20**	72	HGSOC	IIIC G3	WT	WT	Primary	11/12/2013	>1	NACT +IDS (Carboplatin +	CR	YES	48	NED	99	30/04/2022	IHC, GEP, IF

									paclitaxel + Bevacizumab)							
**21**	73	HGSOC	IIIC G3	WT	Altered	Primary	08/03/2018	>10	NACT+IDS (Carboplatin + paclitaxel + Bevacizumab)	PR	YES	2	DOD	12	01/03/2019	IHC, GEP
**22**	32	HGSOC	IV G3	gBRCA1	Altered	Primary	22/03/2018	<0.3	NACT+IDS (Carboplatin + paclitaxel + Bevacizumab)	PR	YES	11	DOD	20	10/12/2019	IHC, GEP, IF
**23**	72	EOvC	IIIC G3	WT	Altered	Primary	29/03/2018	>10	NACT+IDS (Carboplatin + paclitaxel)	CR	NO		NED	45	18/01/2022	IHC, GEP, IF
**24**	58	HGSOC	IIIC G3	WT		Primary	04/06/2018	1	NACT+IDS (Carboplatin + paclitaxel+ Bevacizumab)	PR	YES	5	DOD	22	16/04/2020	IHC, GEP, IF
**25**	73	EOvC	IVB G3	sBRCA1		Primary	17/05/2018	>10	NACT+IDS (Carboplatin + paclitaxel)	CR	YES	7	DOD	29	23/10/2020	IHC, GEP, FC, IF
**26**	80	HGSOC	IVA G3	WT		Primary	27/12/2000	>10	NACT+IDS (Carboplatin + paclitaxel)	PD	YES	1	LOST FUP	1	18/04/2001	IHC, GEP
**27**	82	HGSOC	IIIC G3	WT	WT	Primary	17/05/2018	>10	None	None			DOC	1	20/06/2018	IHC, GEP
**28**	60	HGSOC	IIIC G3	WT	Altered	Primary	29/06/2018	>10	NACT+IDS (Carboplatin + paclitaxel +Bevacizumab)				LOST FUP	2	07/10/2018	IHC, GEP
**29**	63	HGSOC	IIIA2 G3	WT	Altered	Primary	05/07/2018	0	Carboplatin+ Paclitaxel +Bevacizumab	CR	NO		NED	44	02/04/2022	IHC, GEP, FC, IF
**30**	63	HGSOC	IIIC G3	sBRCA2		Primary	07/06/2018	5	Carboplatin + paclitaxel	CR	YES	9	LOST FUP	19	28/01/2020	IHC, GEP, FC, IF
**31**	47	HGSOC	IIIC G3	WT	WT	Primary	12/07/2018	<0.2	Carboplatin+ Paclitaxel+ Bevacizumab	PR	YES	6	DOD	35	28/06/2021	IHC, GEP, FC, IF
**32**	79	OCCC	IVB G3	sBRCA1		Primary	19/02/2019	1	Carboplatin + Paclitaxel +Bevacizumab	PR	YES	16	AWD	31	21/09/2021	IHC, GEP, FC, IF
**33**	70	HGSOC	IIB G3	WT	Altered	Primary	14/08/2018	5	NACT+IDS (carboplatin + Paclitaxel)	CR	YES	6	AWD	44	15/04/2022	IHC, GEP
**34**	61	OCCC	IIIC G3	WT		Primary	20/08/2018	5	Carboplatin + Paclitaxel + Bevacizumab	PD	YES	1	DOD	9	01/06/2019	IHC, GEP, FC, IF
**35**	50	EOvC	IIB G3	WT	WT	Primary	27/08/2018	0	Carboplatin + Paclitaxel	CR	YES	16	AWD	44	30/04/2022	IHC, GEP, FC, IF
**36**	56	OCCC	IIIC G3	WT	Altered	Primary	09/12/2018	>10	NACT + IDS (Carboplatin + Paclitaxel)	LOST						IHC, GEP, FC, IF
**37**	43	HGSOC	IIIB G3	sBRCA1		Primary	13/09/2018	0	Carboplatin + Pegylated doxorubicin	CR	YES	40	AWD	43	30/04/2022	IHC, GEP, FC, IF

**38**	37	HGSOC	IVA G3	WT	Altered	Primary	04/10/2018	<0.2	Carboplatin+ Paclitaxel+ Bevacizumab	CR	YES	14	DOD	31	01/06/2021	IHC, GEP, FC, IF
**39**	42	HGSOC	IVB G3	sBRCA1	Altered	Primary	22/11/2018	0	None	None	YES	6	AWD	28	23/3/2021	IHC, GEP, IF
**40**	74	HGSOC	IIIC G3	WT	WT	Primary	28/11/2018	0.2	Carboplatin + Paclitaxel + Bevacizumab	CR	NO		NED	48	30/04/2022	IHC, GEP, FC, IF
**41**	52	HGSOC	IIIC G3	WT	WT	Primary	14/03/2019	0	Carboplatin + Pegylated doxorubicin	CR	NO		NED	37	30/04/2022	IHC, GEP, FC, IF
**42**	44	HGSOC	IVA G3	sBRCA2		Primary	04/09/2019	>10	NACT+ IDS (Carboplatin Paclitaxel + PARPi)	CR	YES	18	AWD	31	30/04/2022	IHC, GEP, FC, IF
**43**	57	HGSOC	IIIB G3	WT	WT	Primary	15/05/2019	0	Carboplatin + Paclitaxel	CR	NO		NED	35	30/04/2022	IHC, GEP, FC, IF
**44**	65	HGSOC	IIIC G3	WT	WT	Primary	13/06/2019	0.5	Carboplatin + Paclitaxel	LOST						IHC, GEP, FC, IF
**45**	64	HGSOC	IVB G3	WT	Altered	Primary	20/06/2019	0	Carboplatin + Paclitaxel	CR	YES	27	AWD	35	30/04/2022	IHC, GEP, FC, IF
**46**	66	HGSOC	IIIC G3	WT	Altered	Primary	27/06/2019	2	Carboplatin + Paclitaxel + PARPi	PR	YES	30	AWD	35	30/04/2022	IHC, GEP, FC, IF
**47**	74	HGSOC	IIIC G3	WT	WT	Primary	07/11/2019	0.1	Carboplatin + Paclitaxel	CR	YES	6	AWD	35	30/04/2022	IHC, GEP, FC, IF
**48**	56	HGSOC	IVB G3	WT	WT	Primary	24/07/2019	>10	NACT+IDS (Carboplatin+ Paclitaxel+ Bevacizumab)	PD	YES	1	DOD	32	30/03/2022	IHC, GEP, FC, IF

AWD: alive with disease; CR: complete response; DOC: died from other cause; DOD: died of disease; gBRCA: germline and somatic BRCA mutation; FC, flow cytometry; GEP, gene expression profile; HRR: Homologous recombination repair; IHC, immunohistochemistry; IF, immunofluorescence; NACT: neoadjuvant chemotherapy; IDS: interval debulking surgery; NED: non evidence of disease; OS: Overall survival; PARPi: PARP inhibitor; PD: disease progression; PFS1: progression free survival after 1st line; PR: partial response; RT: residual tumour; sBRCA: somatic BRCA mutation; SD: stable disease; WT: Wild type.

Supplementary Table 3. Flow cytometry antibodies

Antigen	Fluorochrome	Clone	Company	Catalogue number
CD3	BUV496	UCHT1	BD Biosciences	564810
CD3	BUV395	SP34-2	BD Biosciences	564117
CD3	APC	UCHT1	BD Biosciences	555335
CD4	Alexa Fluor 700	A161A1	Biolegend	357418
CD8	BUV737	SK1	BD Biosciences	564629
CD14	PerCP	MφP9	BD Biosciences	354786
CD19	PerCP-Cy5.5	SJ25C1	Biolegend	363016
CD33	BV605	P67.6	Biolegend	366612
CD39	PE-Cy7	A1	Biolegend	328212
CD45	PE-Vio770	5B1	Miltenyi Biotec	130-113-119
CD45RA	PE-Cy5.5	MEM-56	Invitrogen	MHCD45RA18
CD48	APC-Vio770	REA426	Miltenyi Biotec	130-106-519
CD62L	BV480	DREG-56	BD Biosciences	566111
CD80	BV650	2D10	Biolegend	305227
CD86	BUV737	2331 (FUN-1)	BD Biosciences	564428
CD95	APC-Vio770	REA738	Miltenyi Biotec	130-113-007
CD137	BV421	4B4-1	Biolegend	309820
CD152 (CTLA-4)	BV605	BNI3	Biolegend	369610
CD155	BV786	TX24	BD Biosciences	744720
CD223 (LAG-3)	APC	REA351	Miltenyi Biotec	130-105-453
CD244 (2B4)	PE-Dazzle594	C1.7	Biolegend	329522
CD274 (PD-L1)	PE	29E.2A3	Biolegend	329706
CD273 (PD-L2)	BV711	MIH18	BD Biosciences	564258
CD279 (PD-1)	BV650	EH12.2H7	Biolegend	329950
CD357 (GITR)	BV711	108-17	Biolegend	371212
CD366 (TIM-3)	PE	REA653	Miltenyi Biotec	130-109-714
EpCAM	DyLight680	323/A3	Novus Biological	NBP2-34532 FR
HLA-DR	BUV395	G46-6	BD Biosciences	565972
HLA-DR	BV480	G46-6	BD Biosciences	566113
KLRG-1	BV785	2F1/KLRG1	Biolegend	138429
TIGIT	BB700	741182	BD Biosciences	747846

Supplementary Table 4. IHC antibodies

Antigen	Clone	Company	Catalogue number	Stainer
**CA-125**	OC125	Cell Marque	CMC26100021	Bench Mark Ultra (Ventana)
**CD3**	2GV6	Ventana	790	Bench Mark Ultra (Ventana)
**CD20**	L26	Leica Biosystems	PA0200	Bench Mark Ultra (Ventana)
**CD163**	MRQ-26	Cell Marque	CMC44370030	Bench Mark Ultra (Ventana)
**LAG-3**	D2G40	Cell Signaling	15372	Discovery ultra (Ventana)
**MUC1**	H23	Ventana	790-4574	Bench Mark Ultra (Ventana)
**NY-ESO-1**	E978	Santa Cruz Biotechnology	Sc-53869	Bench Mark Ultra (Ventana)
**PD-1**	NAT105	Cell Marque	CMC48950041	Bench Mark Ultra (Ventana)
**PD-L1**	22C3	Dako	P03951	Bench Mark Ultra (Ventana)
**TIM-3**	D5D5R	Cell Signaling	45208	Discovery ultra (Ventana)
**WT-1**	6F-H2	Cell Marque	CMC43970031	Bench Mark Ultra (Ventana)

Supplementary Table 5. List of custom Nanostring genes

ADAR1	JUN	SMAD2
ADORA2B	KLRG1	SMAD3
CBLB	MERTK	SMAD7
CCR7	MUC1	SOCS3
CD33	PDCD2	STAT6
CD39	PML-RARA-Tx1	TCF7
CEACAM5	PRDM1	TOX
FOXO1	PROS1	WT1 (alternative)
GAS6	PTPN2	WT1 (canonical)
GATA3	SIRT1	

Supplementary Table 6. IF antibodies

Antigen	Fluorochrome	Clone	Company	Catalogue number	Stainer
**CD39**	RED 610	EPR20627	Abcam	Ab223842	Discovery ultra (Ventana)
**CD137**	FITC	D2Z4Y	Cell Signaling	34594	Discovery ultra (Ventana)

Supplementary Table 8. ROC analysis for the amount of CD137$^+$CD39$^+$ T cells according to the presence of CD137$^+$CD39$^+$PD-1$^+$TIM-3$^+$CD45RA$^-$CD62L$^-$CD95$^+$ cells among CD8$^+$ (A) and CD4$^+$ (B) T lymphocytes

A

	Sensitivity%	95% CI	Specificity%	95% CI
> 0.0500	100	70,1% to 100%	20,0	1,03% to 62,4%
> 0.200	100	70,1% to 100%	60,0	23,1% to 92,9%
> 0.650	100	70,1% to 100%	80,0	37,6% to 99,0%
> 1.25	88,9	56,5% to 99,4%	80,0	37,6% to 99,0%
> 1.75	88,9	56,5% to 99,4%	100	56,6% to 100%
> 2.20	77,8	45,3% to 96,1%	100	56,6% to 100%
> 2.75	66,7	35,4% to 87,9%	100	56,6% to 100%
> 3.70	44,4	18,9% to 73,3%	100	56,6% to 100%
> 5.30	33,3	12,1% to 64,6%	100	56,6% to 100%
> 7.45	22,2	3,95% to 54,7%	100	56,6% to 100%
> 10.6	11,1	0,570% to 43,5%	100	56,6% to 100%

B

	Sensitivity%	95% CI	Specificity%	95% CI
> 1.40	100	67,6% to 100%	16,7	0,855% to 56,4%
> 2.15	100	67,6% to 100%	50,0	18,8% to 81,2%
> 2.75	100	67,6% to 100%	66,7	30,0% to 94,1%
> 4.15	100	67,6% to 100%	83,3	43,6% to 99,1%
> 6.25	100	67,6% to 100%	100	61,0% to 100%
> 9.95	87,5	52,9% to 99,4%	100	61,0% to 100%
> 13.1	75,0	40,9% to 95,6%	100	61,0% to 100%
> 15.3	62,5	30,6% to 86,3%	100	61,0% to 100%
> 18.8	50,0	21,5% to 78,5%	100	61,0% to 100%
> 22.8	37,5	13,7% to 69,4%	100	61,0% to 100%
> 25.5	25,0	4,44% to 59,1%	100	61,0% to 100%
> 26.5	12,5	0,641% to 47,1%	100	61,0% to 100%

The conditions with the best sensitivity and specificity are highlighted in yellow.

Supplementary Table 9. ROC analysis for the amount of CD33$^+$ myeloid cells according to the presence of CD137$^+$CD39$^+$PD-1$^+$TIM-3$^+$CD45RA$^-$CD62L$^-$CD95$^+$ cells among CD8$^+$ (A) and CD4$^+$ (B) T lymphocytes

A

	Sensitivity%	95% CI	Specificity%	95% CI
> 2.30	100	70,1% to 100%	20,0	1,03% to 62,4%
> 7.30	100	70,1% to 100%	40,0	7,11% to 76,9%
> 12.7	100	70,1% to 100%	60,0	23,1% to 92,9%
> 16.1	88,9	56,5% to 99,4%	60,0	23,1% to 92,9%
> 21.6	88,9	56,5% to 99,4%	80,0	37,6% to 99,0%
> 29.2	77,8	45,3% to 96,1%	80,0	37,6% to 99,0%
> 35.8	77,8	45,3% to 96,1%	100	56,6% to 100%

> 43.2	66,7	35,4% to 87,9%	100	56,6% to 100%
> 56.7	55,6	26,7% to 81,1%	100	56,6% to 100%
> 68.2	44,4	18,9% to 73,3%	100	56,6% to 100%
> 74.0	33,3	12,1% to 64,6%	100	56,6% to 100%
> 85.7	22,2	3,95% to 54,7%	100	56,6% to 100%
> 93.7	11,1	0,570% to 43,5%	100	56,6% to 100%

B

	Sensitivity%	95% CI	Specificity%	95% CI
> 2.30	100	67,6% to 100%	16,7	0,855% to 56,4%
> 7.30	87,5	52,9% to 99,4%	16,7	0,855% to 56,4%
> 12.7	87,5	52,9% to 99,4%	33,3	5,92% to 70,0%
> 16.1	75,0	40,9% to 95,6%	33,3	5,92% to 70,0%
> 21.6	75,0	40,9% to 95,6%	50,0	18,8% to 81,2%
> 29.2	75,0	40,9% to 95,6%	66,7	30,0% to 94,1%
> 35.8	75,0	40,9% to 95,6%	83,3	43,6% to 99,1%
> 43.2	62,5	30,6% to 86,3%	83,3	43,6% to 99,1%
> 56.7	50,0	21,5% to 78,5%	83,3	43,6% to 99,1%
> 68.2	37,5	13,7% to 69,4%	83,3	43,6% to 99,1%
> 74.0	25,0	4,44% to 59,1%	83,3	43,6% to 99,1%
> 85.7	12,5	0,641% to 47,1%	83,3	43,6% to 99,1%
> 93.7	0,00	0,00% to 32,4%	83,3	43,6% to 99,1%

The conditions with the best sensitivity and specificity are highlighted in yellow.

Supplementary Figures

Supplementary Figure 1

A

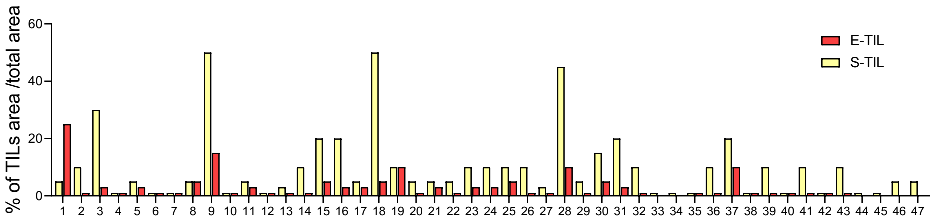

B

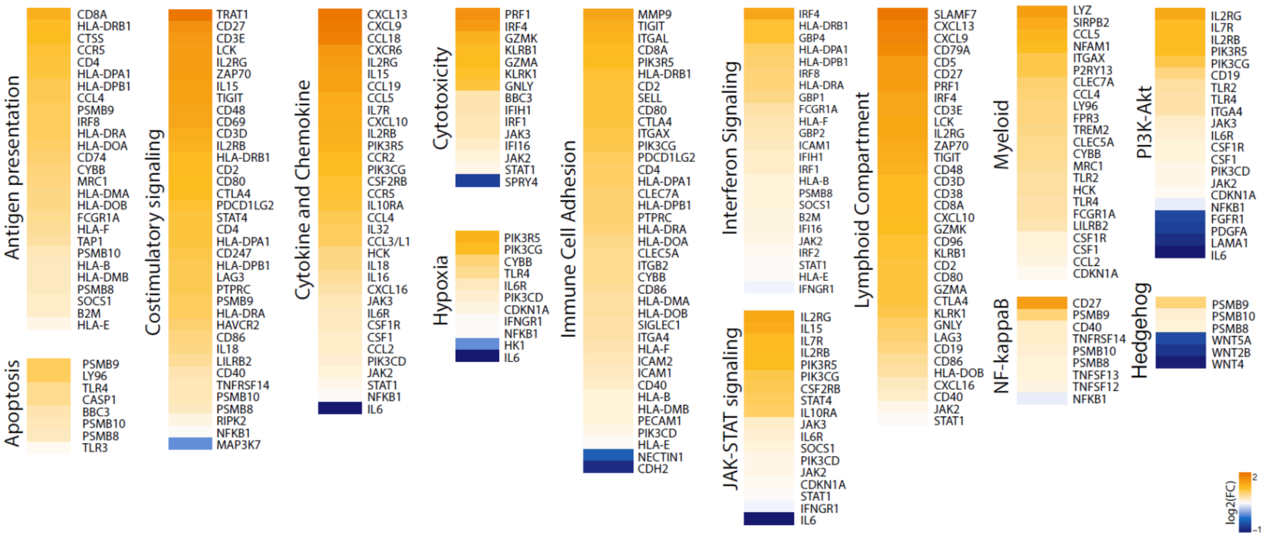

Supplementary Figure 2

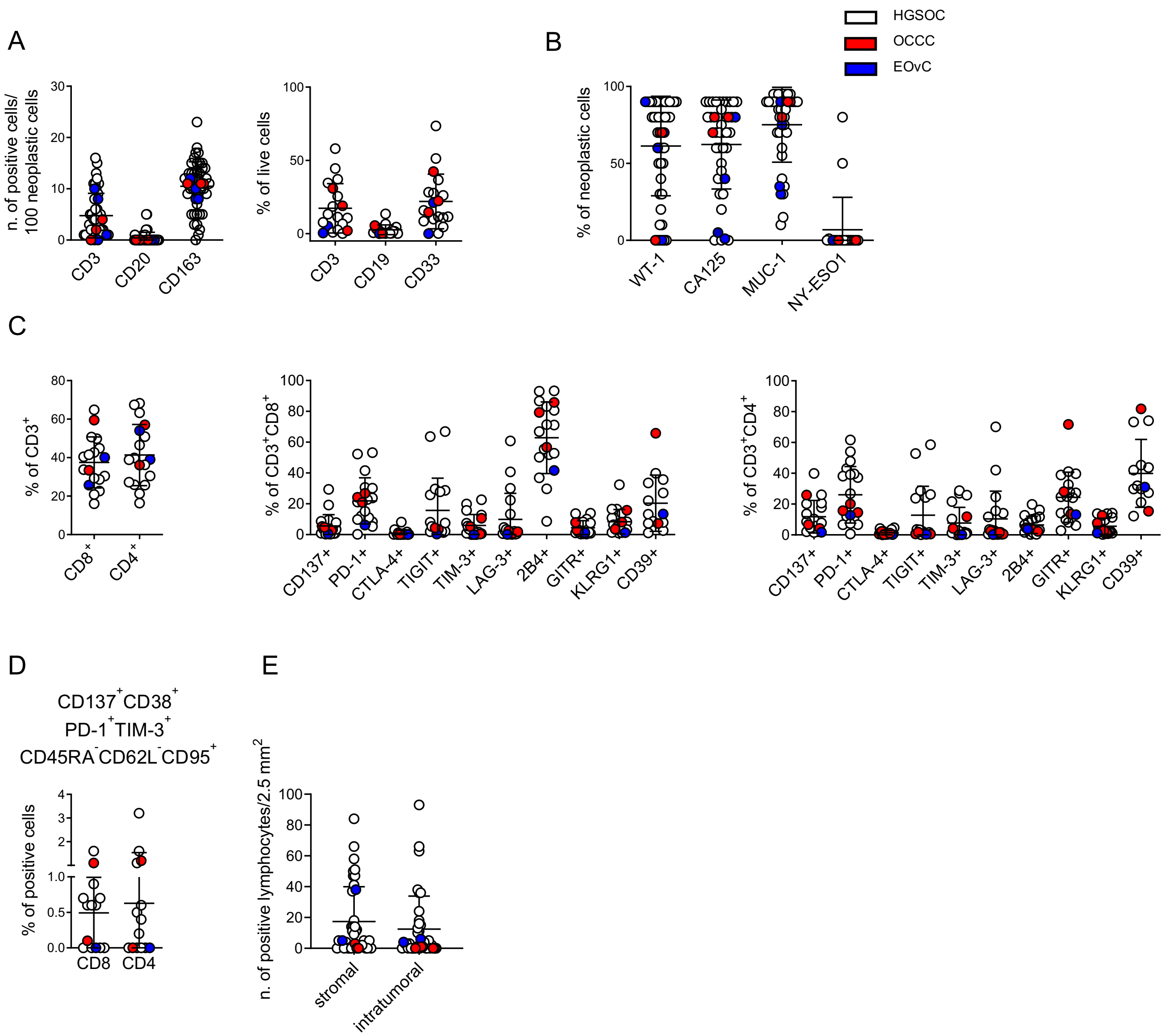

Supplementary Figure 3

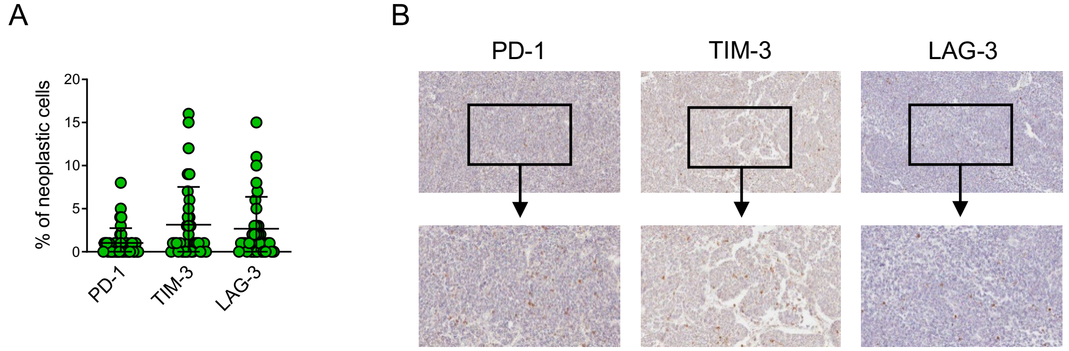

Supplementary Figure 4

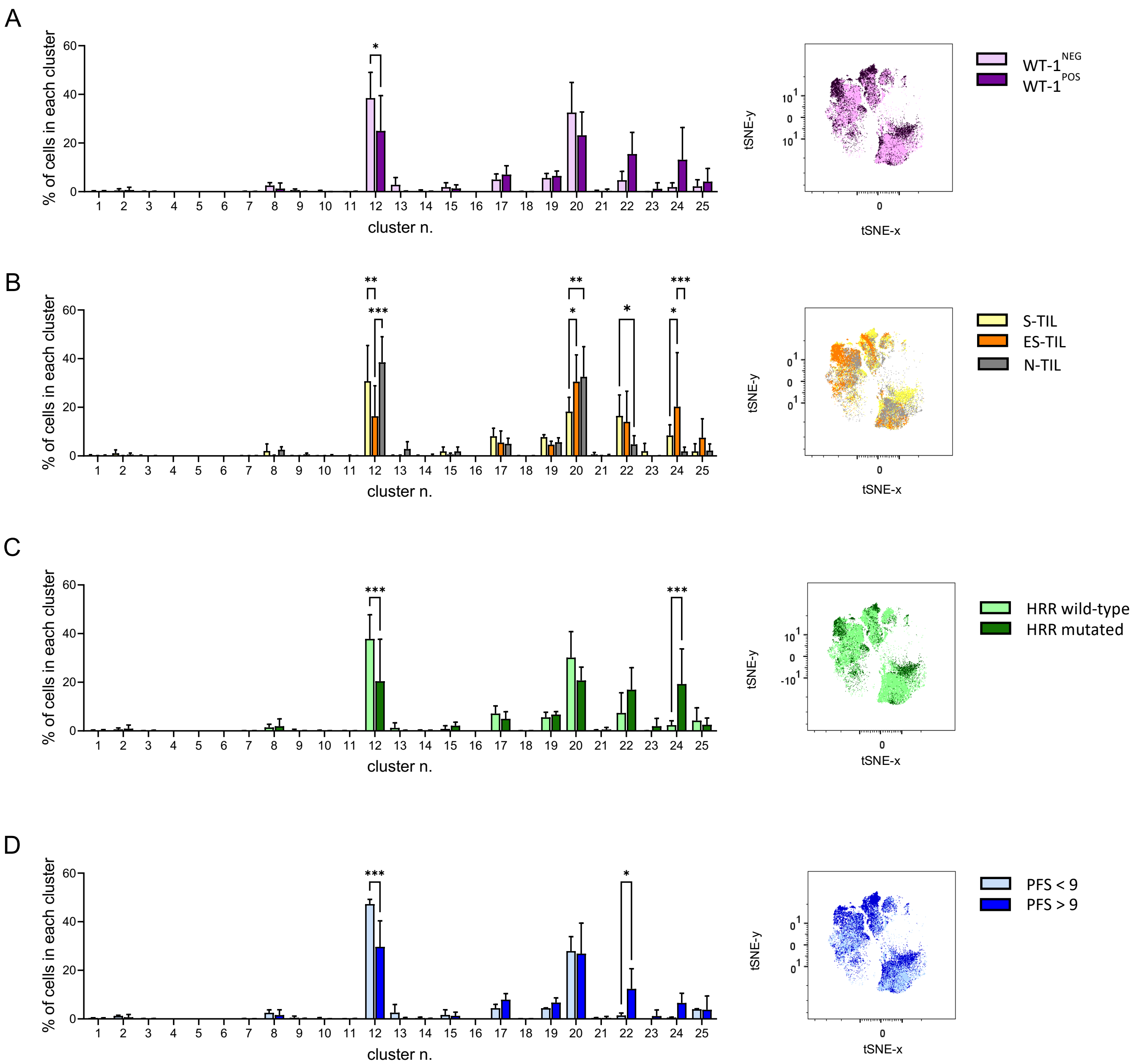

Supplementary Figure 5

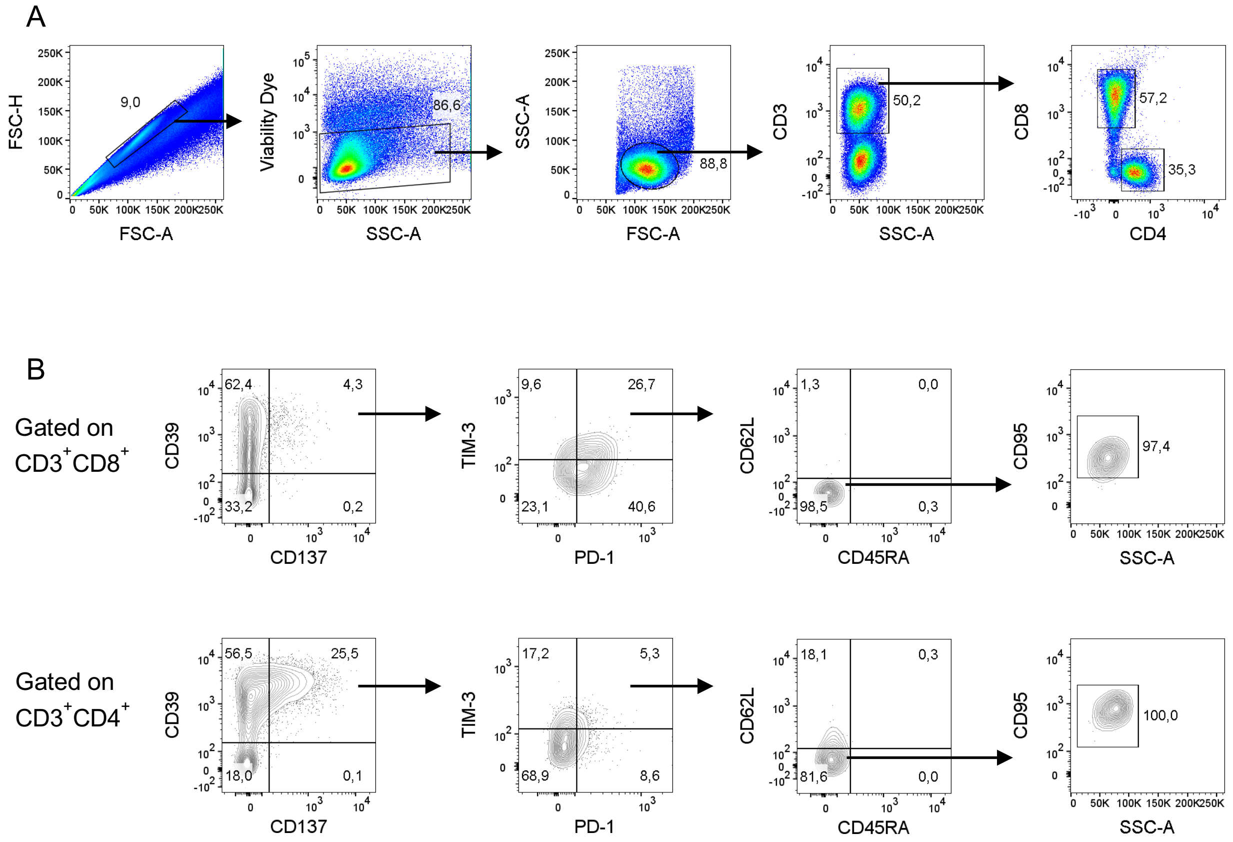

Supplementary Figure 6

A

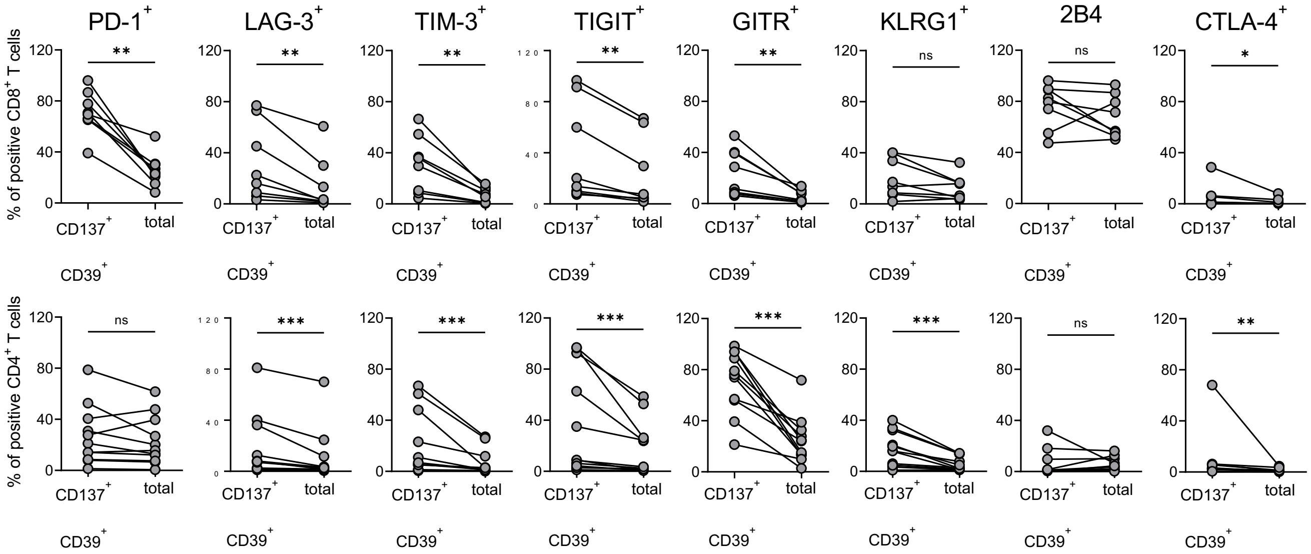

B

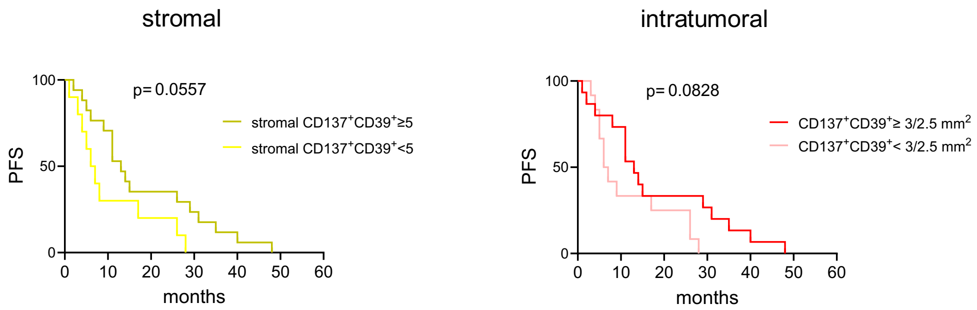

n. at risk

≥5	17	12	6	4	2
<5	10	3	2	0	0

n. at risk

≥3	15	11	5	4	2
<3	12	4	3	0	0

Supplementary Figure 7

A

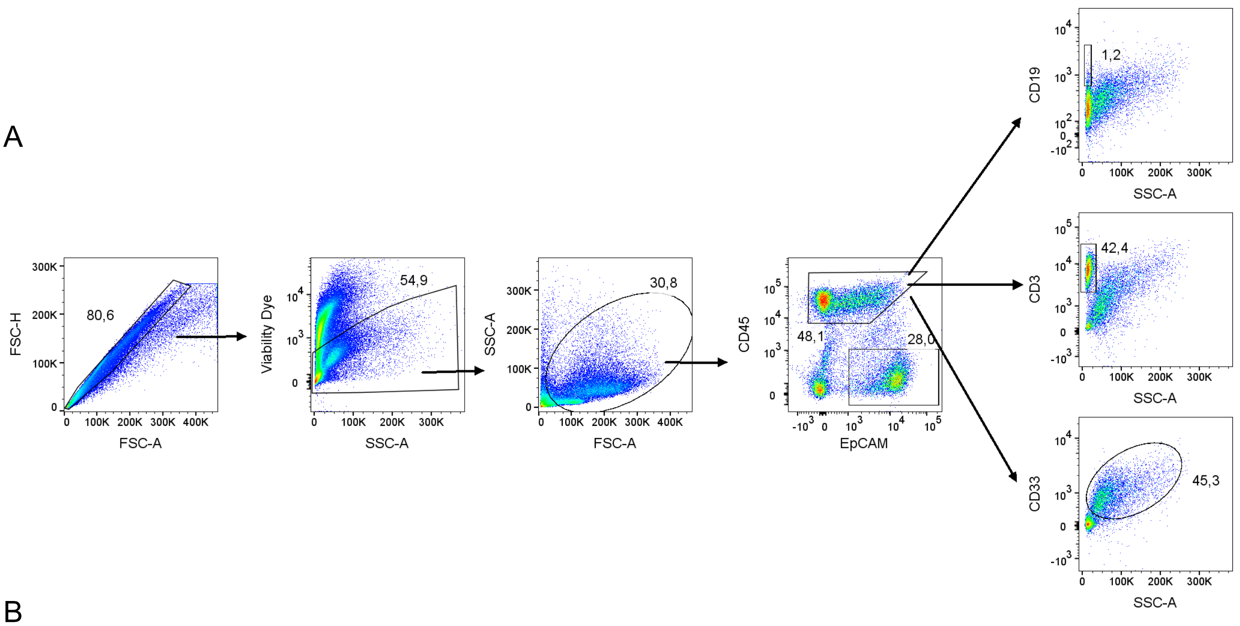

B

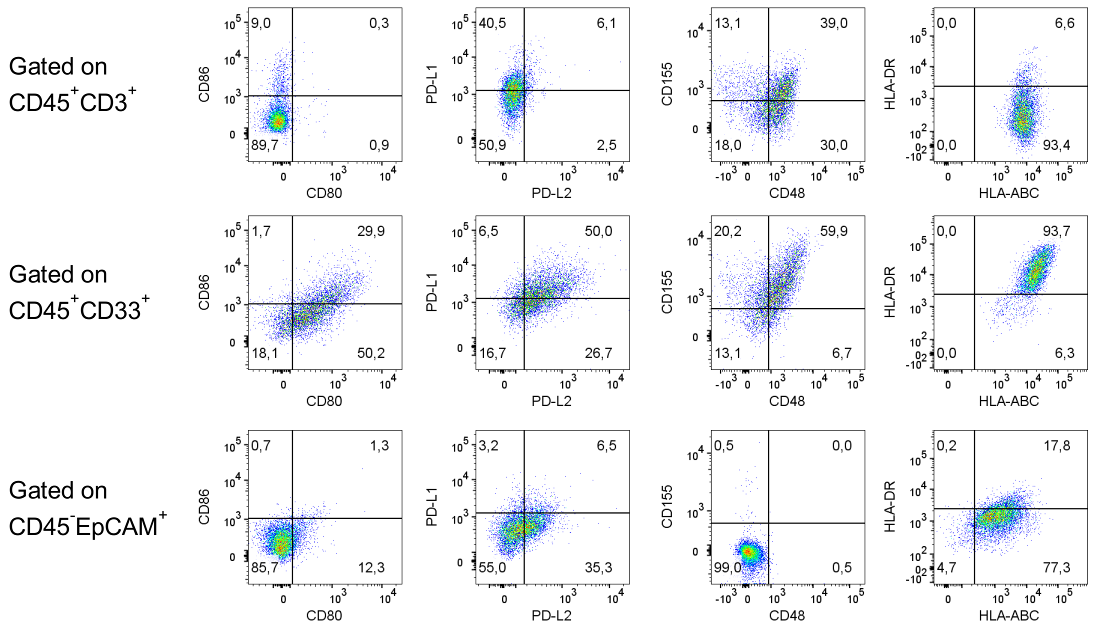

Supplementary Figure 8

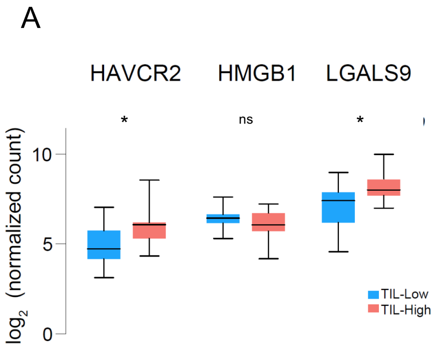

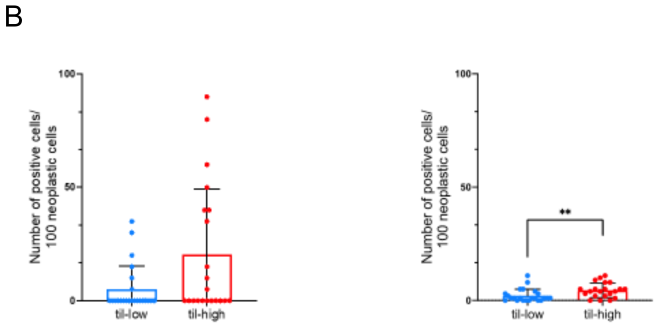

Legends to Supplementary Figures

Supplementary Figure 1. Detail on TILs distribution and genes overexpressed in TIL-High samples. A, quantification for each specimen of TILs in stroma (S-TILs) or epithelium (E-TILs). **B**, heatmaps showing the differential expression of the indicated genes in significant pathways with respect to High-TILs group.

Supplementary Figure 2. Expression of immune-related markers in the different EOC subtypes. The expression of different markers evaluating either composition of immune infiltrate by IHC (**A**, left panel) and flow cytometry (**A**, right panel), TAAs expression (**B**), composition and phenotype of TILs (**C**), presence of T cells with the $CD137^{+}CD39^{+}PD\text{-}1^{+}TIM\text{-}3^{+}CD45RA^{-}CD62L^{-}CD95^{+}$ signature (**D**) or presence of $CD137^{+}CD39^{+}$ lymphocytes by IF in epithelial and stromal compartment (**E**) was evaluated in EOC samples displaying HGSOC (white), EOvC (blue) or OCCC (red) histotypes.

Supplementary Figure 3. IRs expression on TILs by IHC analysis. A, quantification by IHC of lymphoid cells expressing the indicated IRs in 38 (PD-1), 34 (TIM-3) and 33 (LAG-3) EOC specimens. Bars indicate mean values ± SD. **B,** representative stainings for the indicated IRs by x10x(upper panels) and x20 (lower panels) magnification.

Supplementary Figure 4. Differentially expressed clusters in tumor according to biological and clinical variables. Metaclusters frequency (left panels) and overlay (right panels) in n=8 neoplastic samples according to WT-1 expression by tumor cells (**A**), TILs distribution (**B**), mutations in HRR pathway (**C**) and PFS (**D**). Analysis by two-way Anova: * $p<0.05$, ** $p<0.01$, *** $p<0.001$.

Supplementary Figure 5. Representative analysis by manual gating of $CD39^{+}CD137^{+}PD1^{+}TIM3^{+}CD45RA^{-}CD62L^{-}CD95^{+}$ cells among $CD8^{+}$ and $CD4^{+}$ T lymphocytes from a EOC sample. Manual gating for the identification of $CD3^{+}CD4^{+}$ and

$CD3^{+}CD8^{+}$ T lymphocytes (**A**) and of the $CD137^{+}CD39^{+}PD\text{-}1^{+}TIM\text{-}3^{+}CD45RA^{-}CD62L^{-}CD95^{+}$ phenotype in both subpopulations (**B**).

Supplementary Figure 6. Exhaustion markers are enriched in $CD137^{+}CD39^{+}$ T cells compared to total TILs. A, Differential expression of the indicated exhaustion markers by $CD137^{+}CD39^{+}$ and total $CD8^{+}$ (upper panels) or $CD4^{+}$ (lower panels) TILs from n=14 EOC. Analysis by Wilcoxon test: *, $p<0.05$; **, $p<0.01$; ***, $p<0.001$; ns, not significant. **B,** Kaplan-Meier estimates of PFS in 27 patients affected by HGSOC according to the presence of $CD137^{+}CD39^{+}$ lymphocytes above or below the median value for each compartment (5 and 3 / 2.5 mm^2 in stromal and intratumoral compartment, respectively). Lines indicate censored data. Comparisons by Log-rank test. All the patients were treated with platinum-based chemotherapy and in some cases received maintenance therapy (8% with PARP-inhibitors, 29% with bevacizumab, 63% no additional therapy).

Supplementary Figure 7. Representative analysis by manual gating of IR ligands-expressing cells among $CD33^{+}$, $CD3^{+}$ and $EpCAM^{+}$ cells from a EOC sample. Manual gating for the identification of $CD45^{+}CD33^{+}$, $CD45^{+}CD3^{+}$ and $CD45^{-}EpCAM^{+}$ cells (**A**) and for the expression of IR ligands in these subpopulations (**B**).

Supplementary Figure 8. Higher expression of genes encoding for TIM-3 and its ligand Galectin-9 in TIL-High samples. A, boxplots of log2(normalized counts) showing the expression of genes encoding for TIM-3 (HAVCR2) and for its ligands HMGB1 and Galectin-9 (LGALS9) by NanoString analysis in 46 surgical samples separated by TIL percentage into TIL-Low (TIL<8% of tumor cells, n=24) and TIL-High (TIL>8% of tumor cells, n=22) groups. Statistical significance by two-tailed Student's t-test and BH correction: *, $p<0.05$. **B**, Galectin-9 expression assessed by IHC in neoplastic (left panel) and immune cells (right panel), according to TIL percentage (TIL-high vs TIL-low groups). Analysis by Mann-Whitney test: **, $p<0.01$.
